# The effectiveness and acceptability of evidence synthesis summary formats for clinical guideline development groups: a mixed-methods systematic review

**DOI:** 10.1186/s13012-022-01243-2

**Published:** 2022-10-27

**Authors:** Melissa K. Sharp, Dayang Anis Binti Awang Baki, Joan Quigley, Barrie Tyner, Declan Devane, Kamal R. Mahtani, Susan M. Smith, Michelle O’Neill, Máirín Ryan, Barbara Clyne

**Affiliations:** 1 Department of General Practice, RCSI University of Medicine and Health Sciences, 123 St Stephens Green, Dublin 2, Ireland; 2 School of Medicine, Royal College of Surgeons in Ireland, 123 St Stephens Green, Dublin 2, Ireland; 3 Health Information and Quality Authority, George’s Court, George’s Lane, Dublin 7, Ireland; 4 School of Nursing and Midwifery, NUI Galway, Galway, Ireland; 5 Evidence Synthesis Ireland & Cochrane, Galway, Ireland; 6 Nuffield Department of Primary Care Health Sciences, University of Oxford, Oxford, England; 7 Department of Public Health and Primary Care, School of Medicine, Trinity College Dublin, Dublin 2, Ireland; 8 Department of Pharmacology & Therapeutics, Trinity College Dublin, Trinity Health Sciences, James Street, Dublin 8, Ireland

**Keywords:** Presentation of findings, Evidence summaries, Summary of findings table, Communication, Mixed-methods systematic review

## Abstract

**Introduction:**

Clinical guideline development often involves a rigorous synthesis of evidence involving multidisciplinary stakeholders with different priorities and knowledge of evidence synthesis; this makes communicating findings complex. Summary formats are typically used to communicate the results of evidence syntheses; however, there is little consensus on which formats are most effective and acceptable for different stakeholders.

**Methods:**

This mixed-methods systematic review (MMSR) aimed to evaluate the effectiveness and acceptability (e.g. preferences and attitudes and preferences towards) of evidence synthesis summary formats for GDG members. We followed the PRISMA 2020 guideline and Joanna Briggs Institute Manual for Evidence Synthesis for MMSRs. We searched six databases (inception to April 20, 2021) for randomised controlled trials (RCTs), RCTs with a qualitative component, and qualitative studies. Screening, data extraction, and quality appraisal were performed in duplicate. Qualitative findings were synthesised using meta-aggregation, and quantitative findings are described narratively.

**Results:**

We identified 17,240 citations and screened 54 full-text articles, resulting in 22 eligible articles (20 unique studies): 4 articles reported the results of 5 RCTs, one of which also had a qualitative component. The other 18 articles discussed the results of 16 qualitative studies. Therefore, we had 5 trials and 17 qualitative studies to extract data from. Studies were geographically heterogeneous and included a variety of stakeholders and summary formats. All 5 RCTs assessed knowledge or understanding with 3 reporting improvement with newer formats. The qualitative analysis identified 6 categories of recommendations: ‘presenting information’, ‘tailoring information’ for end users, ‘trust in producers and summary’, ‘knowledge required’ to understand findings, ‘quality of evidence’, and properly ‘contextualising information’. Across these categories, the synthesis resulted in 126 recommendations for practice. Nine recommendations were supported by both quantitative and qualitative evidence and 116 by only qualitative. A majority focused on how to present information (*n* = 64) and tailor content for different end users (*n* = 24).

**Conclusions:**

This MMSR provides guidance on how to improve evidence summary structure and layout. This can be used by synthesis producers to better communicate to GDGs. Study findings will inform the co-creation of evidence summary format prototypes based on GDG member’s needs.

Trial registration

The protocol for this project was previously published, and the project was preregistered on Open Science Framework (Clyne and Sharp, Evidence synthesis and translation of findings for national clinical guideline development: addressing the needs and preferences of guideline development groups, 2021; Sharp and Clyne, Evidence synthesis summary formats for decision-makers and Clinical Guideline Development Groups: A mixed-methods systematic review protocol, 2021).

**Supplementary Information:**

The online version contains supplementary material available at 10.1186/s13012-022-01243-2.

Contributions to the literature

- Summaries are often used to communicate evidence synthesis findings; however, there is no consensus on the most effective way to communicate or what works for different audiences.
- This review explored the effectiveness and acceptability of different summary formats for different audiences.
- We identified recommendations to help evidence synthesis producers better communicate to different audiences. These include guidance on formatting, tailoring content for end users, instilling trust in the work, establishing and helping knowledge requirements, detailing the quality of included studies, and properly contextualising findings.
- Results can guide the creation of summary formats better tailored to end user’s needs).

## Background

Clinical guidelines are an important tool for the practice of evidence-based medicine. Often involving rigorous syntheses of the best available evidence, clinical guidelines (CG) aim to improve healthcare in a cost-effective manner by assisting decision-making for clinicians and policymakers [1–3]. Guideline development groups (GDG) are comprised of a multidisciplinary decision-makers such as healthcare professionals, methodologists, and patient representatives. These participants engage in the guideline development process which may involve formal consensus methods amongst these stakeholders. Research on group decision-making within the guideline context indicates that these different stakeholders have different priorities and understandings of knowledge and research evidence [4–6].

In creating guidelines, GDGs need to consider evidentiary factors (such as quality, quantity, and consistency) alongside complex trade-offs between competing benefits and harms, side effects, and risks of various disease management options [7]. The methodological expertise and research knowledge of a GDG can thus influence the quality of a guideline [8] and therefore guideline uptake. Evidence syntheses, such as systematic reviews, may be infrequently used by healthcare managers and policymakers due to intrinsic factors such as format and content and extrinsic factors such as lack of awareness and skills to seek, appraise, and interpret systematic reviews [9, 10]. While for patients involved in guideline development, the strong focus on research evidence can hinder active participation in discussions [11]. Review or evidence synthesis summaries have been proposed as a way to improve the uptake and usefulness of evidence syntheses for decision-makers [9, 10].

Evidence synthesis summaries come in a variety of different formats such as one-page plain language reports, policy briefs, summary of findings tables, visual abstracts or infographics, and more. While summaries may be more easily understandable than complete systematic reviews [12, 13], review summaries are often too long and complex and may require additional work to effectively ‘translate’ the evidence for policymakers [14]. Given the different priorities and knowledge bases of GDG members [4–6], it is reasonable that different stakeholders would have preferences for different formats. Accordingly, research has shown that there is no clear consensus on the most effective way to communicate to all members [12, 13].

It is critical to identify the best summary formats to ensure the best possible communication within multidisciplinary GDGs as they interpret evidence syntheses and develop clinical guidelines to support evidence-based decision-making [15]. This study aimed to evaluate the effectiveness and acceptability of (e.g. preferences for and attitudes towards) different communication formats of evidence synthesis summary formats amongst GDG members. The objectives were as follows: (1) how and to what degree do different summary formats (digital, visual, audio) of presenting evidence synthesis findings impact the end user’s understanding of the review findings? and 2) What are the end users’ preferences for and attitudes towards these formats? To support a multifaceted view on the guideline development process, we conducted a mixed-methods systematic review (MMSR) as this method offers a deeper understanding of findings, more easily identifies discrepancies in the evidence, and is more useful for decision-makers [16, 17]. The MMSR approach also allows one to examine different aspects of a particular phenomenon — i.e. the effects that summary formats may have on knowledge or decision-making and how acceptable these formats were to users [18].

## Methods

We conducted a MMSR according to a preregistered and published protocol [19, 20], following the guidance of the Joanna Briggs Institute (JBI) Manual for Evidence Synthesis, using a convergent segregated approach [17], and the PRISMA 2020 checklist (Additional file 1) [21].

### Study designs and eligibility criteria

Eligible studies were included if they were randomised controlled trials (RCTs) comparing alternative summary formats for evidence syntheses, RCTs with a supplemental qualitative component, or qualitative studies such as focus groups, interviews, or open-ended surveys. Per our protocol, we restricted to these study designs as we chose to focus on the performance and impact of summary formats in optimal settings, and RCTs are the most appropriate design to evaluate effectiveness [20]. We did not include observational studies as there is a high potential that confounding factors will be extensive due to the complexity of stakeholders, evidence synthesis types, and summary formats involved.

Eligible participants were those who could be involved in clinical guideline development groups (e.g. healthcare professionals, policymakers, patient representatives, researchers, methodologists) and outcomes related to effectiveness, acceptability (e.g. views and preferences) of summary formats. We excluded studies involving students, journalists, or the general public as communication to these populations is more complex. Members of the general public were included if they were a patient representative involved in a guideline development group. Use of evidence synthesis summary formats to inform clinicians and patient’s decision-making regarding individual care was not the focus of this review [20].

### Search strategy and study selection

We searched six databases, Ovid MEDLINE, Embase MEDLINE (Medical Literature Analysis and Retrieval System Online), APA (American Psychological Association ) PsycINFO, CINAHL (Cumulative Index to Nursing and Allied Health Literature), Web of Science, and Cochrane Library, from inception to April 20, 2021 (Additional file 2). The search strategy was purposefully sensitive rather than specific. All titles, abstracts, and full texts were independently double screened (DAB, BC, JQ, MKS, BT) using Covidence [22]. Disagreements were discussed between two lead reviewers (BC, MKS) until consensus was achieved. The complete list of eligible articles and potentially relevant studies with exclusion justifications are available on the project’s OSF page [19]. We used the CitationChaser Shiny application to perform backwards citation identification [23, 24]. One reviewer (MKS) manually screened citations that the app was unable to include (e.g. reports without a DOI).

### Data extraction and appraisal of studies

The data extraction form was piloted by two reviewers (MKS, DAB) on one article, required changes were discussed, and the final data extraction was performed using this form and the TiDiER checklist [25]. Study quality was assessed using the JBI Critical Appraisal Checklist for Qualitative Research and the JBI Checklist for RCTs as appropriate [26]. An assessment of the overall certainty of evidence using the GRADE or ConQual approach is not recommended [17, 27] for JBI MMSRs because the data from separate quantitative and qualitative evidence is transformed and integrated. All data extraction was performed independently in duplicate (DAB, BC, JQ, MKS). Disagreements were discussed with the lead author (MKS) and resolved by consensus. The data extraction forms are available on OSF [19].

### Analysis and synthesis of findings

As we did not have a sufficient number of quantitative studies included, we were unable to perform a meta-analysis, the Harboard test for publication bias [28], Egger’s test [29], and statistical heterogeneity [30] as planned. As established in our protocol [20], since we could not perform a meta-analysis, a narrative synthesis was performed.

Qualitative findings were synthesised using the pragmatic meta-aggregation approach which allows a reviewer to present findings of included studies as originally intended by the original authors [31, 32]. Meta-aggregation seeks to enable generalisable statements in the form of recommendations to guide practitioners and policymakers. Findings (defined as a verbatim extract of the author’s analytical interpretation of the results or data) from the “Results” section of manuscripts and accompanying illustrations (direct quotations or statements from participants) were coded as ‘unequivocal evidence’. Findings with no illustrations or an illustration lacking clear association were ‘equivocal/credible’. Findings which were not supported by the data were ‘unsupported’. Interpretations of the study results given by the study authors were not coded to avoid interfering with the transformation and integration process in an MMSR when combining the quantitative and qualitative evidence [31, 33].

**Fig. 1:**
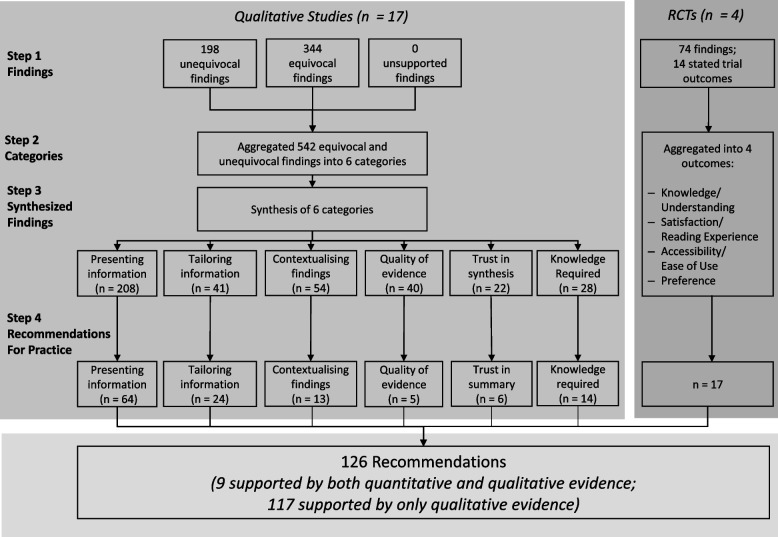
Mixed Methods Synthesis Steps and Results

**Fig. 2:**
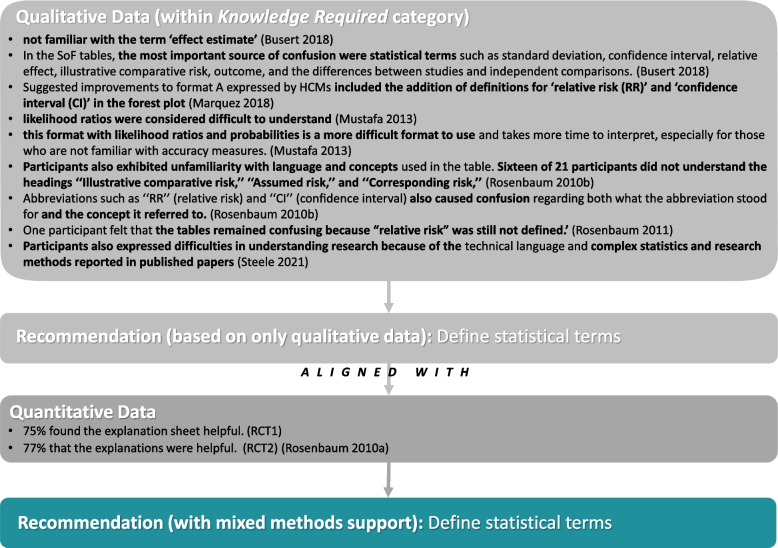
Qualitative Synthesis Example

NVivo 12 was used to analyse results from primary qualitative studies and accompanying illustrations [34]. One author (MKS) performed the initial line-by-line coding of equivocal, unequivocal, and unsupported findings which was checked by a second reviewer (BC) [17, 35]. MKS is a mixed-methods researcher with a background with psychoepidemiology and metaresearch, whereas BC is a health services researcher who has extensive experience in evidence synthesis and working with guideline development groups. These findings were then synthesized into categories, based on similarity in meaning. Categories were proposed by MKS, reviewed by BC, and refined through discussions. All findings were double coded to categories by both reviewers, and MKS distilled the findings into actionable recommendations for practice which were then reviewed by BC. As recommended by JBI, we did not differentiate between equivocal and unequivocal findings when aggregating them into categories. These coding steps are detailed in Fig. 1, and an example of the late-stage synthesis steps is in Fig. 2.

To synthesise findings from both qualitative and quantitative evidence, we followed the JBI guidance for MMSR and used a convergent segregated approach as we conducted separate quantitative and qualitative syntheses and then integrated the findings of each [17, 36]. We juxtaposed the synthesised quantitative and qualitative findings and then organized the linked findings in a single line of reasoning to produce an overall configured analysis [18]. This integration process identifies areas of convergence, inconsistency, or contradiction [37]. The final table of recommendations was agreed upon through discussion by the entire multidisciplinary author team. Since overall assessments of the certainty of evidence using the GRADE or ConQual approach are not recommended for MMSRs, we created a cutpoint (supported by ≥ 3 evidence streams) as a blunt proxy for level of evidence to create a more usable set of recommendations.

## Results

### Search results

**Fig. 3:**
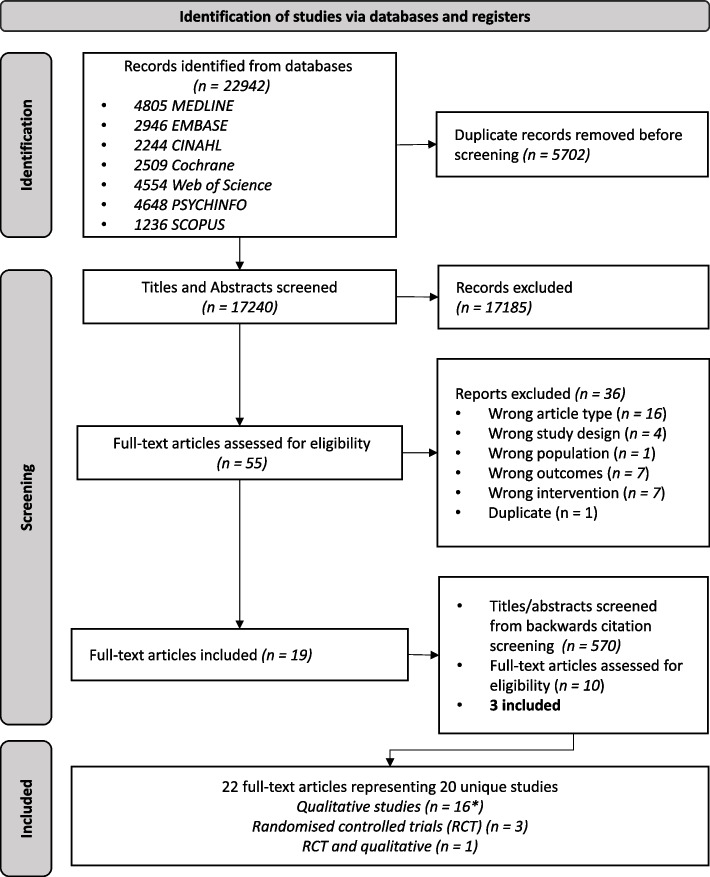
PRISMA Flow Diagram

After deduplication of identified records, we screened 17,240 titles and abstracts, the majority of which were excluded (*n* = 17,185). The yield rate is slightly lower than previous estimates likely due to the breadth of stakeholders, summary formats, and outcomes of interest [38, 39]. We reviewed 54 full-text articles and identified 22 articles for inclusion which all underwent backwards citation screening (Fig. 3). The search strategy output and reasons for inclusion/exclusion files are available on OSF [19]. Of note, many studies had multiple phases or participant groups. We included the study if we could clearly separate the methods and results for the phase and/or group. Where possible, we extracted information only from the eligible phase/group.

### Characteristics of included studies

Our final sample included 22 full-text articles representing 20 unique studies. This included 16 qualitative studies, 4 RCTs, and 1 mixed-methods RCT and qualitative study (Tables 1 and 2) involving 908 total participants from a variety of different stakeholder groups (Table 1). Many studies involved a multidisciplinary mix of participants such as researchers, health professionals, and policymakers [40, 41, 43–45, 47–50, 54–56, 59–61], although some had homogenous groups of clinicians [51, 52, 57] or decision-makers [42, 46, 53]. The majority of types of evidence syntheses were systematic reviews, but one study related specifically to network meta-analyses (NMA), one to diagnostic test accuracy (DTA) reviews, and one to updating reviews. Seven studies involved an international mix of participants [42, 48, 53, 54, 58, 60, 61], five were from Canada [43, 46, 47, 51, 52], three from the USA [44, 45, 49, 55, 56], two from Croatia [41, 59], two from England [40, 57], and one from Kenya [50]. Most were funded by national agencies [41–43, 45–47, 49, 51, 52, 55, 56, 59] such as the Canadian Institutes of Health Research [43, 47, 51, 52] or the Agency for Healthcare Research and Quality [45, 46, 49, 55, 56].

**Table 1:** Included qualitative studies

Author (year, country)	Collection method	Participants	Format(s) evaluated	Topics
Babatunde *(2018, England)* [40]	Semi-structured interviews *(and questionnaires)*^b^	*N* = 21. Clinicians (11), researchers (5), epidemiologists (3), health service/trial managers (2)	Evidence flowers and summary table	Musculoskeletal conditions
Buljan *(2020, Croatia)* [41]	Focus groups	*N* = 20. Patient advocates (9), doctors (4), *medical students (7)*^*a*^	Plain language summary, infographic, scientific abstract	Breech presentation
Busert *(2018, International)* [42]	Semi-structured interviews	*N* = 18. Public-health decision-makers	4-page summary with Summary of findings (SoF) table and Grading of Recommendations, Assessment, Development and Evaluations (GRADE) ratings	Food, alcohol, and tobacco portion/packaging
Dobbins *(2004, Canada)* [43]	Focus groups	*N* = 46. Medical officers (7), programme managers/coordinators (25), decision-makers (14)	Summary statement	Tobacco control
Hartling *(2016, 2017, USA)* [44, 45]	Semi-structured interviews	*N* = 8. Guideline developers (3), healthcare providers (3), research funders (1), health insurers (1)	‘Rapid products’ (evidence inventory, rapid response, rapid review)	Venous thromboembolism
Hartling *(2018, Canada)* [46]	Semi-structured interviews	*N* = 6. Decision-makers	3-page summary	Youth mental health
Marquez *(2018, Canada)* [47]	Semi-structured interviews *(and survey)* ^b^ Semi-structured interviews	*N* = 11. Healthcare managers (5), policymakers (6) *N* = 12. Healthcare managers (5), policymakers (7)	Summary prototype	Healthcare management/services
Mustafa *(2015, International)* [48]	Semi-structured interviews, workshop discussions	*N* = 20. Researchers, health professionals, guideline developers	3 formats^c^ of GRADE evidence tables	Diagnostic test accuracy reviews
Newbery *(2013, USA)* [49]	Focus groups *(with questionnaires)* Individual feedback *(and questionnaires)*	*N* = 15. Health insurer (2), insurance/former policymaker (2), clinicians (3), researchers (2), governmental research directors (2), research consultant (1) *N* = 3. Community physicians	7 differently formatted executive summaries	Acute otis media
Opiyo *(2013, Kenya)* [50]	Semi-structured interviews	*N* = 16. Multidisciplinary guideline development group members	SoF tables, graded-entry summary, normal systematic review	Newborn care, hand hygiene
Perrier *(2014, Canada)* [51]	Focus groups	*N* = 10. Family physicians	Case-based and evidence-based prototypes	Rosacea
Perrier *(2014, Canada)* [52]	Focus groups	*N* = 32. Primary care physicians	Two summary prototypes	Rosacea
Rosenbaum *(2011, International)* [53]	Semi-structured interviews	*N* = 18. Policymakers and managers	Short summaries	Healthcare management/services
Rosenbaum *(2010, International)* [54]	Semi-structured interviews (and workshops)	*N* = 21. Health professionals, researchers	SoF tables	Deep vein thrombosis
Smith, Totten *(2019, USA)* [55, 56]	Semi-structured interviews	*N* = 6. Department director (1), health system experts (4), guideline developers (2)	MAGICapp, Tableau	Chronic pain
Steele *(2021, England)* [57]	Semi-structured interviews	*N* = 7. Mental health clinicians	One-page summary, full systematic review	Mental health
Yepes-Nunez *(2019, International)* [58]	Semi-structured interviews	*N* = 32. Methodologists (21), meta-analysis users (5), clinicians (6)	SoF tables	Network meta analyses

^a^Population and accompanying RCT not eligible. ^b^Not eligible. ^c^There were 4 formats in total, but only 3 were shown to the user testing group. Abbreviations: United States of America (*USA*), Germany, Austria, and Switzerland (*DACH*), summary of findings (*SoF*), Grading of Recommendations, Assessment, Development and Evaluations (*GRADE*), *MAGIC* Making GRADE the Irresistible Choice)

**Table 2:** Included randomised controlled trials

Author (year, country)	Participants	Intervention and comparators	Primary (***secondary***) outcomes and operationalization (number of questions, type, scales)	Focus
Buljan (2018, Croatia) [59]	*N* = 163 (eligible across trials) 99 patient representatives, 64 doctors *(171 students)*^*a*^	Infographic, plain language summary, scientific abstract (doctors only)	Understanding/knowledge (10, open ended) *Reading experience (5, summative, 10-point scale)* *User-friendliness (5, summative, 10-point scale)*	Breech presentation
Carrasco-Labra (2016, International) [60]	*N* = 284 Health professionals (122), guideline developers (42), researchers (120)	2 versions (1 existing, 1 alternate) of Grading of Recommendations, Assessment, Development and Evaluations (GRADE), Summary of Findings (SoF) tables	Understanding (7, multiple choice, 5-point *scale*) *Accessibility of information (3, 7-point scale; 1, 5-point scale)* *Satisfaction (6, yes/no)* *Preference (1, 7-point scale)*	Paediatric probiotics
Opiyo (2013, Kenya) [50]	*N* = 70 Paediatricians (32), medical/nursing officers (18), researchers (5), healthcare trainers (5), governmental/clinical officers (7), pharmacists (2), administrator (1)	3 different topic ‘evidence packs’ 1. Normal systematic review (SR) 2. SR plus SoF tables 3. Graded-entry SR	Understanding (2 per format, 3-point scale) *Composite endpoint (1, 5-point scale)* *Clarity (1 per format, 3-point scale)* *Accessibility (2 per format, 5-point scale)*	Hand hygiene, newborn care, newborn feeding regimens
Rosenbaum (2010, International) [61]	*N* = 72 (RCT1) Healthcare professionals *N* = 33 (RCT2) Staff from Cochrane entities	Normal Cochrane review (CR) with no SoF table CR with SoF table (limited formatting) CR with SoF table (full formatting) Normal Cochrane review (CR) with no SoF table CR with SoF table (revised)	User satisfaction (unclear, multiple choice) Perceived understanding and ease of use *(7, 8-point scale)* Understanding (4, unclear) Time spent finding key results (1, continuous)	Deep vein thrombosis

^a^Population does not meet eligibility criteria for this review. ^c^Sixty-five participants completed the questionnaires. Group membership details are given for these 65, not the full 70 enrolled in the study. Abbreviations: Summary of findings (*SoF*), Grading of Recommendations, Assessment, Development and Evaluations (*GRADE*), Cochrane review (*CR*)

The TiDiER checklist was used to gather intervention data detailed in Tables 1, 2, and 3. The majority of included qualitative studies conducted either focus groups [41, 43, 49, 51, 52] or one-on-one semi-structured interviews [40, 42, 44–47, 50, 53–58, 62] (Table 1). RCTs were conducted either with an online survey [59, 60] or through in-person workshops (Tables 2 and 3) [50, 61]. There were a wide variety of summary formats tested including de novo summary prototypes [43, 46, 47, 49–53, 57], Grading of Recommendations, Assessment, Development and Evaluations (GRADE) Summary of Findings (SoF) evidence tables [42, 48, 50, 54, 58], MAGICapp [55, 56], Tableau [55, 56], evidence flowers [40], plain language summaries [41], and infographics [41]. Summary formats covered a wide variety of clinical topics (Tables 1 and 2).

**Table 3:** Description of interventions in randomised controlled trials

Author (year)	Brief description of intervention	Intervention location, mode of delivery, time limit	Materials and components
Buljan (2018, Croatia) [59]	Infographic, plain language summary, scientific abstract	Online, electronic, none	Participants read one of the summary formats, followed by the survey (first a numeracy test with sufficient delay for the knowledge test). Patient representatives were presented with the infographic or PLS, and doctors were presented all three formats
Carrasco-Labra (2016, International) [60]	Summary of findings table	Online, electronic, 25 mi	Participants were exposed to one table containing either the new or current format, and the outcomes understanding, accessibility of information, satisfaction, and preference were assessed. Participants were then shown the table to which they were not initially allocated, and their preference was assessed
Opiyo (2013, Kenya) [50]	Evidence summaries in 3 formats (A, B, C)	In-person workshop, paper, 45 min	Summaries were delivered to participants as prereading materials 1 month before the workshop. Participants completed questionnaires on the first day of the guideline development workshop before the panel discussions about guidance recommendations
Rosenbaum (2010, International) [61]	RCT1: Normal Cochrane review (CR) with no SoF table CR with SoF table (limited formatting) CR with SoF table (full formatting) RCT2: Normal Cochrane review (CR) with no SoF table CR with SoF table (revised)	RCT1: In person workshop, unclear, unclear RCT2: In-person workshop, unclear, unclear	Participants first answered a questionnaire based on the version of the review they had received. Then, all participants were shown both formatting versions of the SoF tables and were instructed to answer a final set of questions measuring their preferences and attitudes about the inclusion summary of findings table in reviews

Abbreviations: Summary of findings (*SoF*), Cochrane review (*CR*)

### Quality appraisal

We found the quality of reporting for the qualitative studies was quite poor (Additional file 3). The main weakness across these studies included not providing information on philosophical perspectives (11/17) [40, 41, 43–47, 49–51, 53, 55, 56], not locating the researcher culturally or theoretically (15/17) [40–42, 46–54, 56–58], and not addressing the influence of the researcher on the research (15/17) [40–42, 44–56, 58]. Several interviews or focus groups also did not provide clear direct quotes from participants (6/17) [43, 47, 49, 51, 55, 56, 62]. On the other hand, the four quantitative studies were mostly reported clearly with low risk of bias [50, 59–61]. The main weaknesses is related to descriptions of the blinding of treatment assignment for the outcome assessors and those delivering treatment (2/4) [50, 61].

### Quantitative analysis

The summary formats tested across the five included RCTs (described across four papers) are described in detail in Table 3. Four RCTs compared alternative versions of SoF tables against a format in current practice and/or a standard systematic review [50, 60, 61] One study compared an infographic to a plain language summary (PLS) and scientific abstract (SA) [59]. Studies were largely multidisciplinary, and results were not presented by stakeholder group. An exception to this was the study by Buljan et al. (2018) which conducted separate trials with patient representatives (‘consumers’) and doctors. There were no differences between the groups in knowledge scores for both the plain-language summary (PLS) and infographic formats. However, patient representatives reported lower satisfaction (user-friendliness) and reading experience with both formats when compared to doctors. As the quantitative studies used a variety of scales and summary formats, we could only summarise results narratively.

In preparation for the mixed-methods synthesis, we identified 74 individual findings from quantitative studies (Additional file 4) and synthesised these into four main areas which related to review outcomes of Knowledge/Understanding, Satisfaction/Reading Experience, Accessibility/Ease of Use, and Preference (Fig. 1). These individual findings helped identify areas of convergence, inconsistency, or contradiction with the qualitative findings and recommendations described later.

### Knowledge or understanding

All five RCTs assessed knowledge or understanding as an outcome (Table 4). No studies employed standardised measures, choosing to use study-specific questions. Two articles, reporting the results of three studies, found that the new format improved knowledge or understanding [60, 61]. Carasco-Labra et al. reported that compared to a standard SoFs table, a new format of SoF table with seven alternative items improved understanding [60]. Of seven items testing understanding, three showed similar results, two showed small differences favouring the new format, and two (understanding risk difference and quality of the evidence associated with a treatment effect) showed large differences favouring the new format [63% (95% *CI*: 55, 71) and 62% (95% *CI*: 52, 71) more correct answers, respectively]. In two small RCTs, Rosenbaum et al. found that the inclusion of a SoF table in a review improved understanding and rapid retrieval of key findings compared to reviews with no SoF table [61]. In the second RCT, there were large differences in the proportion that correctly answered questions about risk in the control group (44% vs. 93%, *P* = 0.003) and risk in the intervention group (11% vs. 87%, *P* < 0.001). Two studies reported no significant differences between formats in knowledge or understanding [50, 59].

**Table 4:** Quantitative results

Author (year) interventions	Primary (***secondary***) outcome measures	Results			
		Understanding/knowledge	Satisfaction/reading experience	Accessibility/ease of use	Preference
Buljan (2018) [59] • Infographic • PLS • SA (doctors only)	Understanding/knowledge (max score = 10) *Reading experience (max score = 50)* *User-friendliness (max score = 50)*	Patients (*n* = 99), median score (95% CI) Infographic: 7.0 (6.0–7.0) *PLS*: 7.0 (6.0–7.0) *P* = 0.511 Doctors (*n* = 64), median score (95% CI) Infographic: 8.0 (6.0–8.0) *PLS*: 8.0 (7.0–9.0) *SA*: 8.0 (5.9–9.0) *P* = 0.611 Significant predictor of knowledge score • Patients only: awareness of Cochrane SRs (*OR* 5.3; 95% *CI*: 1.7–16.6), 13.4% of variance	*Reading experience* Patients (*n* = 99), median score (95% CI) Infographic: 33.0 (28.0–36.0) *PLS*: 22.5 (19.0–27.4) *P* < 0.001 Doctors (*n* = 64), median score (95% CI) Infographic: 37.0 (26.8–41.3) *PLS*: 32.0 (30.0–39.9) *SA*: 24.0 (21.3–27.2) *P* = 0.002	*User-friendliness* Patients (*n* = 99), median score (95% CI) Infographic: 30.0 (25.5–34.5) *PLS*: 21.0 (19.0–25.0) *P* < 0.001 Doctors (*n* = 64), median score (95% CI) Infographic: 36.0 (30.9–40.0) *PLS*: 29.0 (26.8–36.2) *SA*: 25.0 (23.5–27.2) *P* = 0.003	Not reported
Carrasco-Labra (2016) [60] • Existing GRADE SoF table • Alternate GRADE SoF tables	Understanding (7 multiple-choice questions on 5-point scale, analysed at question level) *Accessibility of information (5-point scale), satisfaction (6 yes/no questions analysed at question level), preference (7-point scale)*	1. 4/7 items risk difference (RD, 95% CI) in favour of alternate SoF tables 2. Understanding of quality of evidence and treatment effect *RD*: 62% (52–71), *P* < 0.001 3. Ability to determine risk difference *RD*: 63% (54.6–71), *P* < 0.001 4. Ability to quantify risk *RD*: 6% (0.1–13.3), *P* = 0.06 5. Understanding of quality of evidence *RD*: 7% (0.1–12.4), *P* = 0.06 6. 3/7 items similar results (RD 95% CI) between formats 7. Ability to interpret risk *RD*: 0% (−5.3–5.4), *P* = 0.99 8. Ability to relate N of participant/studies and outcomes *RD*: −3% (−7.5–1.7), *P* = 1.00 9. Ability to interpret footnotes *RD*: 7% (−2–15), *P* = 0.18	Questions where largest proportion in favour of alternate SoF tables: 5/6 Questions where largest proportion in favour of existing SoF table: 1/6	Overall accessibility mean difference (MD (SE)) in favour of alternate SoF: *MD* 0.3 (0.11), *P* = 0.001	MD (SE) in favour of alternate *SoF*: 2.8 (1.6)
Opiyo (2013) [50] • Normal systematic review (SR) • SR plus SoF tables • Graded-entry SR	Understanding (2 questions per format on 3-point scale) *Composite endpoint (on 5-point scale)* *Clarity (1 question per format on 3-point scale)* *Accessibility (2 questions per format on 5-point scale)*	Odds ratio (OR) (95% CI) SR plus SoF versus SR, *OR* 0.59 (0.32–1.07) Graded-entry SR versus SR, *OR* 0.66 (0.36–1.21) Sub-group analyses: policymakers understanding • SR plus SoF *OR* 1.5 (0.15–15.15) • Graded-entry *OR* 1.5 (0.64 t–3.54)	Not reported	*Accessibility* SR plus SoF versus SR • OR (95% CI) 0.91 • (0.57–1.46) • MD (95% CI) 0.11 (−0.71–0.48) Graded-entry SR versus SR • *OR* 1.06 (1.06 to 2.20) • - MD (95% CI) 0.52 (0.06–0.99)	Not reported
Rosenbaum (2010) [61] RCT 1 • Cochrane review (CR) with no SoF table • CR with SoF table (limited formatting) • CR with SoF table (full formatting)	User satisfaction (multiple-choice questionnaire) Perceived understanding and ease of use *(7 questions on 8-point scale)*	Proportion who agree/strongly agree main findings were easy to understand % (95% CI) • No SoF table: 56 (37–75) • With SoF table (both formats): 60 (46–74) • *P* = 0.54	Not reported	Proportion who agree/strongly agree very accessible % (95% CI) • No SoF table: 17 (2–32) • With SoF table (both formats): 41 (27–56) • *P* = 0.037	65% agreed CR should include SOF with the proposed format
Rosenbaum (2010) RCT 2 • Cochrane review (CR) with no SoF table • CR with SoF table (limited formatting) • CR with SoF table (full formatting)	Understanding (4 questions) Time spent finding 5 key results	2/4 difference in proportion of questions correctly answered (%, 95% CI) favouring with SOF 1. Risk in the control group: 44% (21–67) versus 93% (81–100), *P* = 0.003) 2. Risk in the intervention group: 11% (0–26) versus 87% (69–100), *P* < 0.001) 2/4 no difference in proportion of questions correctly answered (%, 95% CI) 1. Confidence of review authors: without SoF 67% (45–88) vs. with SoF 87% (69–100), *P* = 0.18 2. Identifying important outcomes: without SoF 33% (9–57) versus with SoF 53% (28–79), *P* = 0.27	Not reported	Mean time (min) finding answers Risk in the control group • No SoF table: 4 • With SoF table: 1.5 • *P* = 0.02 Risk in the intervention group • No SoF table: 2.8 • With SoF table: 1.3 *P* = 0.118 Confidence of review authors • No SoF table: 1.5 • With SoF table: 2.1 • *P* = 0.47 Identifying important outcomes • No SoF table: 1.9 • With SoF table: 2.0 • *P* = 0.88	84% agreed CR should include SOF with the proposed format

*Abbreviations*: *CI* Confidence interval, *CR* Cochrane review, *GRADE* Grading of Recommendations, Assessment, Development and Evaluations, *MD* Mean difference, *NR* Not reported, *OR* Odds ratio, *PLS* Plain language summary, *RD* Risk difference, *SA* Scientific abstract, *SD* Standard deviation, *SE* Standard error, *SoF* Summary of findings, *SR* Systematic review

### Ease of use/accessibility

All five RCTs provided some assessment of ease of use and accessibility, measured in a variety of ways (Table 4). Buljan et al. reported that user-friendliness was higher for an infographic compared to a PLS for doctors and patient representatives [patients median infographic score: 30.0 (95% *CI*: 25.5–34.5) vs. *PLS*: 21.0 (19.0–25.0); doctors median infographic score: 36.0 (30.9–40.0) vs. *PLS*: 29.0 (26.8–36.2)] [59], while Carasco-Labra et al. reported that in six out of seven domains, participants rated information in the alternative SoF table as more accessible overall (*MD* 0.3, *SE* 0.11, *P* = 0.001) [60]. Opyio et al.’s graded-entry SoF formats were associated with a higher mean composite score for clarity and accessibility of information about the quality of evidence (adjusted mean difference 0.52, 95% *CI*: 0.06 to 0.99) [50]. In two small RCTs, Rosenbaum et al. found that participants with the SoF format were more likely to respond that the main findings were accessible [61]. The second RCT demonstrated, that in general, participants with the SoF format spent less time finding answers to key questions than those without.

### Satisfaction

Two studies assessed satisfaction (Table 4). Buljan et al. reported that both patients and doctors rated an infographic better for reading experience than a PLS, even though it did not improve knowledge [patients median infographic score: 33.0 (95% *CI*: 28.0–36.0) vs. *PLS*: 22.5 (19.0–27.4); doctors median infographic score: 37.0 (26.8–41.3) vs. *PLS*: 24.0 (21.3–27.2)] [59]. Carasco-Labra et al. reported that participants were more satisfied with the new format of SoF tables (5/6 questions where the largest proportion was in favour of alternate SoF tables) [60].

### Preference

Two studies assessed user preference (Table 4). Carasco-Labra et al. reported that participants consistently preferred the new format of SoF tables (*MD* 2.8, *SD* 1.6) [60]. Similarly, Rosenbaum et al. reported that overall participants preferred the alternative (or new) format of SoF tables compared to the current formats (*MD/SD*: 2.8/1.6) [61].

### Qualitative analysis

**Fig. 4:**
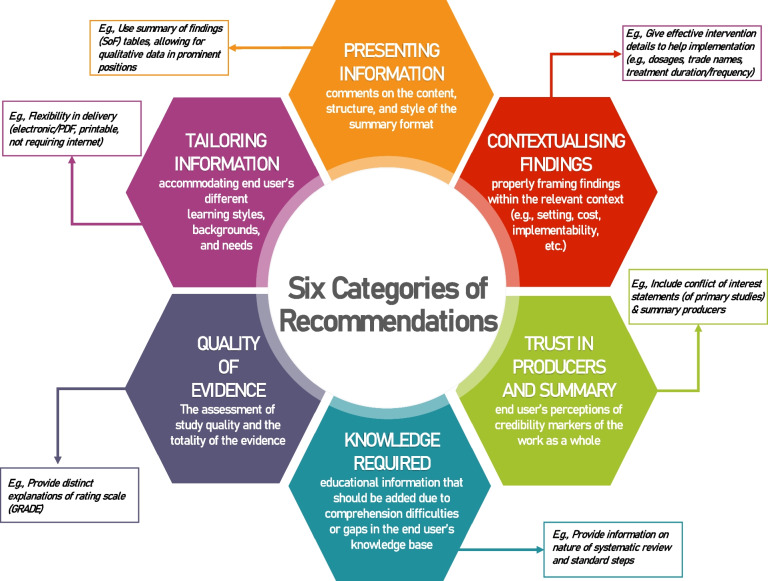
Categories of Recommendations

From 16 qualitative studies and 1 RCT with a supplemental qualitative component, line by line coding identified 542 equivocal and unequivocal findings within the “Results” section of the articles. No unsupported findings were identified (Fig. 1). From these initial 542 findings, we synthesized them further into 393 findings across 6 categories defined as follows (Fig. 4):

1. Presenting information (comments on the content, structure, and style of the summary format)
2. Tailoring information (inherently linked to the presentation of information but more focused on accommodating end user’s different learning styles, backgrounds, and needs to appropriately tailoring content)
3. Contextualising findings (properly framing the findings themselves within the relevant context by providing information such as setting, cost constraints, and ability to implement findings)
4. Trust in producers and summary (end user’s perceptions of credibility markers of the work as a whole — such as transparency, funding sources, and clear references — i.e. that the work was rigorously done by qualified individuals)
5. Quality of evidence (focused on the assessment of study quality and the totality of the evidence including how assessments were reached and information about rating)
6. Knowledge required to understand findings (educational information that should be added to summaries due to comprehension difficulties or gaps in end user’s knowledge base)

These 393 synthesized findings were then reviewed again by two authors (MKS, BC) to produce 126 recommendations for practice which, where possible, are presented based on targeted GDG members (Additional files 5 and 6) and specific type of evidence syntheses such as NMA (*n* = 22), DTA reviews (*n* = 2), and updating reviews (*n* = 8). A total of 94 recommendations could broadly apply to broader types of evidence synthesis. As previously mentioned, most studies contained diverse multidisciplinary participants. When quotes from participants were reported, it was often not attributed to a specific stakeholder, and several studies also included no direct quotes from participants. However, where possible, recommendations are presented according to group membership (noted by superscripts). The individual 126 recommendations from the qualitative synthesis are available in Additional file 5, alongside the citation(s) which support each, whether they also had mixed-methods support, and which end user may have expressed the recommendation.

A majority of recommendations are related to presenting information (*n* = 64) or Tailoring Information for the end user (*n* = 24). For example, items under the presenting information category include things like ‘*use bullet points*’, ‘*flag important information by bolding/highlighting*’, use ‘*greyscale-friendly colours*’, and ‘*avoid abbreviations*.’ Tailoring Information included guidance on how to create bespoke customised documents with ‘*easily extractable information to forward to colleagues*’ and the importance of ‘*clarifying the audience*’ that the report is for and about. Several items regarding the presentation of numerical and statistical findings were identified across several categories. For example, for Presenting Information, it was suggested to ‘*use absolute numbers, not probabilities*’ and *to* ‘*decrease numeric/statistical data*’, whereas the contextualising findings category suggested ‘*interpretation aids for statistics*’ and noted that policy/decision-makers are ‘not interested in methodology. The Knowledge Required category highlighted the lack of awareness of abbreviations, recommending to ‘*avoid abbreviations* (*e.g. RR for relative risk, CI for confidence intervals*)’ altogether. Some of these items are intrinsically linked as the Knowledge Required recommendations highlighted that for readers, certain items like ‘*forest plots are difficult to understand*’, so providing ‘*interpretation of statistical results*’ and ‘*defining statistical terms*’ can be helpful.

### Mixed-methods synthesis

The four outcome areas for the quantitative evidence (e.g. Knowledge, Satisfaction) were also covered by the qualitative evidence. However, due to the large heterogeneity in stakeholders, formats, and assessments methods, it was difficult to determine whether the qualitative evidence helped explain differences in size or direction of effects in the quantitative studies.

From 74 individual quantitative findings (Additional file 4), we identified 17 which converged with at least one of the 126 qualitative recommendations (Additional file 5). Some of these 17 items supported the same recommendation (e.g. several findings supported the use of summary of findings tables), so in total, these 17 quantitative findings supported 9 qualitative findings. Some of these items are inherently linked as SoF tables (4) are often using the GRADE rating scale (8). Similarly, the items about assessments of quality (7 and 9) likely to refer to GRADE as well. The 9 recommendations with mixed-methods support are marked with an asterisk in Figs. 6, 7, and 8 (Additional file 6) and include providing a clear summary report as follows:

1. Is structured
2. Is brief
3. Provides information on the standard steps and nature of the review
4. Presents results in summary of findings (SoF) tables
5. Defines statistical terms
6. Provides interpretations of statistical results
7. Includes assessments of quality
8. Describes the rating scale (GRADE)
9. Describes how authors arrived at their assessments of quality

Throughout our recommendations, there are items which may appear at face value to be contradictory. However, they simply accommodate different learning styles (e.g. ‘*use summary of findings tables*’ and ‘*use narrative summaries*’); thus, these are considered complimentary. Relatedly, there were some items that were expressed by different groups which echoed the end user’s different needs. For example, the ‘*Abstract Methods Results and Discussion (AMRaD) format*’ was advocated by clinicians, whereas ‘*avoid academic formatting*’ was expressed by policy/decision-makers. Additionally there are some items that are similar but were expressed for very different purposes — for example ‘*including author’s names*’ is in both the presenting information and trust in producers and summary categories as some participants flagged this as a clear indicator of their trust in the quality of the work, whereas others just wanted the information for general factual transparency purposes (Additional file 6: Figs. 6, 7, 8).

**Fig. 5:**
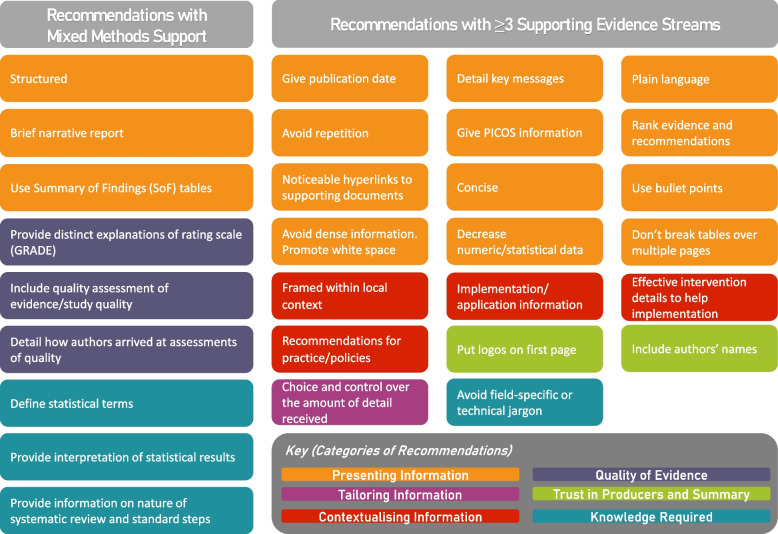
Recommendations with Mixed Methods or at least 3 supporting evidence streams

As an overall aim of a MMSR is to provide actionable recommendations, in an effort to strike a balance between 9 recommendations with mixed-methods support and 94 recommendations from the qualitative literature, we reviewed all recommendations (Additional file 5) and took a pragmatic approach to narrow down the list to those with three or more studies supporting them (or mixed-methods support) (Additional file 7). Using this approach, there were the aforementioned 9 recommendations with mixed-methods support and 20 recommendations with supporting evidence from three or more studies (Fig. 5). Most of the recommendations were from the Presenting Information category (*n* =12), e.g. ‘*give publication date*’, ‘*use bullet points*’, and ‘*detail key messages*’. Three were focused on contextualising information (e.g. ‘*framed within local context*’, ‘*effective intervention details to help implementation*’), two were on Trust in producers and Summary (e.g. ‘*put logos on first page*’, ‘*include author’s names*’), one was from the knowledge required category (e.g. ‘*avoid field-specific or technical jargon*’), and one was from the Tailoring information category (e.g. ‘*choice and control over the amount of detail received*’).

## Discussion

This mixed-methods systematic review synthesised the evidence on the effectiveness and acceptability of different evidence synthesis summary formats. The quantitative results suggest that alternative versions of SoF tables compared to a current format and/or a standard systematic review improved knowledge or understanding. However, assessments of study quality revealed that half of the included trials had poor reporting related to the blinding of outcome assessors and those delivering treatment. There was insufficient evidence to establish a ‘gold-standard’ summary format amongst end users; however, qualitative studies offered a wealth of data such that we could synthesize findings into 126 actionable recommendations across six thematic areas. Thirty-two of the 126 recommendations were for specific types of reviews (e.g. NMA, DTA, and updating reviews). Ninety-four items could be broadly applied to a variety of evidence synthesis types, and nine had mixed-methods support. A further 21 of the actionable recommendations were also supported by at least three different studies, a proxy measure adopted to indicate items with a larger evidence base. These 30 recommendations can be used to promote more effective communication with different stakeholders. To help with potential implementation, we also delineated findings by review type and stakeholder group where possible as there was some evidence that end user’s had different preferences.

The interventions included in our review were diverse with a variety of outcome measures. The majority of studies tested de novo summary prototypes, making it difficult to draw comparisons. However, five studies assessed GRADE SoF tables, and a significant portion of our recommendations pertain to summary of findings tables and GRADE ratings. In fact, there were enough findings concerning the quality assessment of studies and use of the GRADE scale that it warranted its own category ‘Quality of Evidence’ in the final recommendations. Previous work focused on US National Guidelines Clearinghouse clinical practice guidelines published between 2011 and 2018 found that the GRADE scale was inconsistently used, and only 1 in 10 (7/67, 10.4%) guidelines explicitly reported consideration of all criteria to assess the certainty in the evidence [63]. As reflected in three of our nine recommendations with mixed-method support, GRADE is an important factor in evidence summary formats. Recent work has highlighted that there are many improvements to be made in terms of consistency in presenting GRADE symbols and explaining the recommendations [64]. This aligns with seven articles in our review which supported the need to be explicit about how the scale is used, recommending to ‘*provide distinct explanations of rating scale (GRADE).*’ Four studies also supported detailing ‘*how authors arrived at assessments of quality*’ (Additional file 5). Many included interventions tended to be in a traditional academic style in that they were largely text based. Accordingly, numerous recommendations addressed how to ‘*flag important*’ and ‘*avoid dense information*’ through ‘*structured*’, ‘*brief*’, and ‘*concise*’ formats with ‘*prominent subheadings*’. Many recommendations such as ‘*including quality assessments of evidence/study quality*’, ‘*provide distinct explanations of rating scale*’, ‘*choice and control over the amount of detail received*’ and ‘*structured*’ information with ‘*intervention details to help implementation*’ are also aligned with several items on the dissemination checklist for Cochrane reviews [65].

The need for structured presentation of information is also supported by previous work. Brandt et al. found that 181 internal medicine and general practice physicians had a clear preference for multi-layered guideline presentation formats [66]. Short menu formats and visual aids have been shown to improve performance when participants are presented with both conditional probability and natural frequency formats [67]. One study found that, across different levels of object numeracy and education, fact boxes (i.e. simple tabular messages) were more engaging than normal text. They also led to more comprehension and slightly more knowledge recall after 6 weeks compared to the same information in text [15].

Other than MAGICApp and Tableau, no other interactive summary formats were identified in our review. Furthermore, no studies that used audio-visual strategies such as podcasts or videos were identified in this review. There is some evidence that video abstracts are more effective than graphical abstracts and traditional abstracts in comprehension, understanding, and reading experience [68]. Audio summaries also show some promising results. University staff listening to a podcast summary of a Cochrane review had the highest rates of comprehension in comparison with those who read a plain language summary or abstract [69]. Future research should explore and test these formats with GDG members.

Many general tenets were supported by multiple studies involving multidisciplinary stakeholders. For example, concerns about the presentation of numerical and statistical results resulted in recommendations across several of our categories. Similar to our findings, Cochrane’s plain language expectations for authors of cochrane summaries (PLEACS) standards recommend presenting numerical information in terms of absolute effects and as natural frequencies [70]. A 2017 meta-analysis also supported the use of natural frequencies. Their study found that performance rates when interpreting natural frequencies increased to 24% compared to only 4% when presented in a probability format. However, three-quarters of participants still failed to obtain the correct solution with either presentation [67]. On the other hand, a 2020 study by Buljan et al. found that numerical presentation (and framing) had no effect on consumer’s and biomedical student’s understanding of health information in plain language summaries [71]. Previous research established that the required literacy for even plain language summaries is higher (over 10 to 15 years of education) than the recommended US 6th grade (11 or 12 years old) reading level [72]. All of this prior work reinforces the idea that effective interactions with evidence synthesis summaries require certain baseline knowledge. This review has provided specific knowledge areas to address as detailed in the Knowledge Required category (e.g. the need to define terms, explain methodologies, grading scales, and statistics and generally provide a supplemental explanation sheet to end users). Initiatives such as the International Guideline Development Credentialing and Certification Program (INGUIDE) [73] may also help address some of these knowledge needs by ensuring that guideline development group members have the necessary competencies.

Our recommendations are proposals for consideration, not strict rules for practice, especially considering that the evidence base supporting many recommendations is weak, and not all may be practical for resource-limited teams. The nine recommendations with mixed-methods support could be considered as essential for any summary format producer, with the additional 20 items with 3 or more evidence streams supporting them as desirable considerations. However, the included studies that these recommendations are based on often did not discuss time or resources required to actually produce the summary format(s) which could make implementation difficult. For example, inclusion of certain items, particularly those related to ‘contextualising findings’, may require additional work or expertise which some may consider to be outside the scope of a typical review [53]. However, these suggestions should not be ignored as research has shown that context is rarely provided in sufficient detail in existing reviews and guidelines [74], and applying evidence synthesis findings to local contexts is a major weakness reported by some health technology assessment (HTA) units trying to promote healthcare decision-making [75].

The strengths of this study include the mixed-methods approach and an extensive search strategy. However, our study has several limitations. Firstly, we did not include observational studies, although during screening we excluded few studies based on their study design (Fig. 3) [76]. The main limitations of our findings relate to the issues of completeness of the reporting of included studies. Several articles did not provide a copy or access to the summary format(s) tested so it was sometimes difficult to properly contextualise their results. Additionally, it was often difficult to attribute a finding to a specific stakeholder group as included studies often did not provide group membership details about quotes used. This meant that many of our recommendations are non-specific as we were unable to fully decipher what works for who and under which circumstances. Stakeholders involved in guideline development have different styles of reasoning and knowledge bases to draw from [6]; therefore, drawing conclusions that are stakeholder group specific is complex. Even within one group (e.g. patient representatives), one size does not fit all when presenting recommendations [77]. However, we recommend that future work with multidisciplinary stakeholders should denote group membership when reporting quotes from participants as this was a deficit in our included studies. For example, while there is some reporting guidance for what public or patient version of clinical guidelines should include [78], we are still missing a step in the process, wherein it is unclear what works best for patient representatives involved in clinical guideline development groups. Lastly, we excluded studies in the general population and students. Studies have shown that PLS improved understanding in these populations [79, 80].

## Conclusions

Our results provide valuable information that can be used to improve existing formats and inform future research aimed at developing more effective evidence synthesis summary formats. The nine recommendations with mixed-methods support can be considered essential to consider for any summary format producer. The additional 20 items with 3 or more evidence streams supporting them can be considered as desirable, with further exploration needed into the full set of 126 items. Future research should further explore these proposed recommendations amongst the different guideline development group members to explore which items are particularly important for which stakeholder. Our research team plans to conduct a prioritisation exercise for these recommendations so we can use them as guidance for focus group workshops with GDG members. Furthermore, other mediums of summary formats not identified in this review could be explored further such as the use of podcasts or video abstracts or summaries.

## Supplementary Information

**Additional file 1.** PRISMA checklist.

**Additional file 2.** Search strategy results.

**Additional file 3.** Data extraction workbook.

**Additional file 4.** Quantitative findings.

**Additional file 5.** Qualitative synthesis recommendations.

**Additional file 6:** Figures 6, 7, and 8. Recommendations for Practice.

**Additional file 7.** Qualitative synthesis recommendations (with at least 3 supporting studies or mixed methods support).

## Data Availability

The study was previously preregistered on the Open Science Framework, and the protocol was published in HRB Open Research [19, 76]. The datasets generated and/or analysed during the current study are available on Open Science Framework (OSF). Data are available under the terms of the Creative Commons Attribution 4.0 International license (CC-BY 4.0).
