# Supplementary material for: The effectiveness and acceptability of evidence synthesis summary formats for clinical guideline development groups: a mixed-methods systematic review
Source: Implement Sci. 2022 Oct 27;17:74. doi: 10.1186/s13012-022-01243-2 (PMC9615384; doi:10.1186/s13012-022-01243-2)
Supplement: Supplementary file 2 — Additional file 2. Search strategy results. [file 13012_2022_1243_MOESM2_ESM.docx]

**Appendix 2.** Search strategy results

|  | **Ovid MEDLINE(R) and Epub Ahead of Print, In-Process, In-Data-Review & Other Non-Indexed Citations, Daily and Versions(R) 1946 to April 20, 2021** |  |
| --- | --- | --- |
| 1 | Exp Administrative Personnel/ | 41028 |
| 2 | ((health OR healthcare OR hospital*) ADJ2 (administrator* OR analyst* OR decisionmak* OR decision-mak* OR manager* OR official* OR policymak* OR policy-mak* OR policy OR policies OR provider)).tw. | 73693 |
| 3 | exp Decision Making/ OR exp Health Policy/ OR exp Policy Making/ | 333117 |
| 4 | ((decision* OR policy OR policies) ADJ2 (analys* OR analyz* OR maker* OR making OR develop*)).tw. | 214272 |
| 5 | (analyst* OR clinician OR decision-mak* OR decisionmak* OR doctor OR guideline development group* OR advisory group OR knowledge user* OR knowledge-user* OR policy-mak* OR policymak* OR stakeholder* OR stake-holder* OR stake holder* OR end user* OR end-user*).tw. | 360402 |
| **6** | **1 OR 2 OR 3 OR 4 OR 5** | 732659 |
| 7 | Exp Evidence Based Practice/ OR exp "Review Literature as Topic"/ OR exp Meta-analysis as topic/ OR exp Systematic reviews as topic/ OR exp Technology Assessment, Biomedical/ | 130525 |
| 8 | (knowledge ADJ2 synthes*).tw. OR exp Health Knowledge, Attitudes, Practice/ | 117395 |
| 9 | (meta*) ADJ2 (analysis OR regression OR review OR overview OR synthes*) | 252187 |
| 10 | meta-analy* OR meta-regression OR meta-review* OR meta-synthes* OR megasynthes* | 232485 |
| 11 | (evidence) ADJ2 (synthes* OR summar*) | 20942 |
| 12 | (quantitative OR qualitative OR systematic OR rapid OR scoping OR realist OR Cochrane OR evidence) ADJ2 (review* OR overview*) | 271217 |
| 13 | HTA OR (health technology assessment) | 6758 |
| **14** |  |  |
| 15 | exp Data Visualization/ OR exp Health communication/ OR exp Implementation science/ OR exp Information Dissemination/ | 21119 |
| 16 | (summary of findings) OR summary-of-findings OR table* OR tabular | 156300 |
| 17 | plain-language summar* OR (plain language summar*) | 1791 |
| 18 | infographic* OR podcast* OR visual abstract* OR fact box* OR summary format OR blogshot OR blog shot OR podcast OR video OR GRADE evidence profile OR policy brief OR league table* OR bulletin OR infogram or 1-page summary OR SUPPORT summary OR brief* or summar* OR graphic* OR audio | 1013255 |
| 19 | (communicat* OR presentat*) ADJ2 (finding*) | 3502 |
| **20** | **15 OR 16 OR 17 OR 18 OR 19** | 1174260 |
| 21 | perceive OR understand OR understanding OR acceptability OR effectiveness OR efficacy OR satisfaction OR usability | 2708622 |
| 22 | usefulness OR credibility OR clarity OR comprehensive OR appeal OR appropriateness OR preference$ | 694443 |
| 23 | 21 OR 22 | 3253556 |
| **24** | **6 AND 14 AND 20 AND 23** | 4805 |

|  | **EMBASE** |  |
| --- | --- | --- |
| 1 | 'administrative personnel'/mj | 9141 |
| 2 | ((health OR healthcare OR hospital*) NEXT/2 (administrator* OR analyst* OR decisionmak* OR decision-mak* OR manager* OR official* OR policymak* OR policy-mak* OR policy OR policies OR provider)) | 370399 |
| 3 | Decision Making/exp OR (Policy NEXT/1 Making) OR (Health NEXT/1 Policy) | 587540 |
| 4 | ((decision* OR policy OR policies) NEXT/2 (analys* OR analyz* OR maker* OR making OR develop*)) | 533941 |
| 5 | (analyst* OR clinician OR decision-mak* OR decisionmak* OR doctor OR ‘guideline development group*’ OR ‘advisory group’ OR ‘knowledge user*’ OR knowledge-user* OR policy-mak* OR policymak* OR stakeholder* OR stake-holder* OR ‘stake holder*’ OR ‘end user*’ OR end-user*) | 927433 |
| **6** | **1 OR 2 OR 3 OR 4 OR 5** | 1257534 |
| 7 | 'evidence based practice'/exp OR ‘Review Literature as Topic’ OR ‘meta-analysis as topic’ OR ‘Systematic reviews as topic’ OR 'biomedical technology assessment'/exp | 1442231 |
| 8 | (knowledge NEXT/2 synthes*). OR (Health NEXT/2 Knowledge NEXT/2 Practice) | 1098 |
| 9 | (meta*) NEXT/2 (analysis OR regression OR review OR overview OR synthes*) | 350215 |
| 10 | meta-analy* OR meta-regression OR meta-review* OR meta-synthes* OR megasynthes* | 336567 |
| 11 | (evidence) NEXT/2 (synthes* OR summar*) | 9183 |
| 12 | (quantitative OR qualitative OR systematic OR rapid OR scoping OR realist OR Cochrane OR evidence) NEXT/2 (review* OR overview*) | 419635 |
| 13 | (HTA OR ‘health technology assessment’):ti,ab,kw | 11162 |
| **14** | **7 OR 8 OR 9 OR 10 OR 11 OR 12 OR 13** | 1634632 |
| 15 | 'data visualization'/exp OR 'implementation science'/exp OR (information NEXT/1 dissemination) OR (health NEXT/2 information NEXT/2 communication) | 24453 |
| 16 | ‘summary of findings’ OR summary-of-findings OR table$ OR tabular | 529799 |
| 17 | ‘plain language summar*’ | 357 |
| 18 | (infographic* OR podcast* OR ‘visual abstract*’ OR ‘fact box*’ OR blogshot OR ‘blog shot’ OR podcast OR video OR ‘GRADE evidence profile’ OR ‘policy brief’ OR ‘league table*’ OR bulletin OR infogram OR brief OR briefing$ OR summary OR summaries OR graphic* OR audio):ti,ab,kw | 853836 |
| 19 | (communicat* OR presentat*) NEXT/2 (finding*) | 4142 |
| **20** | **15 OR 16 OR 17 OR 18 OR 19** | 1383518 |
| 21 | (perceive OR understand OR understanding OR acceptability OR effectiveness OR efficacy OR satisfaction OR usability):ti,ab,kw | 3518446 |
| 22 | (usefulness OR credibility OR clarity OR comprehensive OR appeal OR appropriateness OR preference$):ti,ab,kw | 877449 |
| 23 | 21 OR 22 | 4205267 |
| **24** | **6 AND 14 AND 20 AND 23** | **7160** |
| 25 | 24 is limited to Embase only records, excluding Medline records n = 2946 | 2946 |

|  | **CINAHL** |  |
| --- | --- | --- |
| 1 | 'administrative personnel' | 18716 |
| 2 | ((health OR healthcare OR hospital*) N2 (administrator* OR analyst* OR decisionmak* OR decision-mak* OR manager* OR official* OR policymak* OR policy-mak* OR policy OR policies OR provider)) | 353762 |
| 3 | (MM "Decision Making, Organizational") OR (MH "Policy Making") | 17023 |
| 4 | ((decision* OR policy OR policies) N2 (analys* OR analyz* OR maker* OR making OR develop*)) | 196106 |
| 5 | (analyst* OR clinician OR decision-mak* OR decisionmak* OR doctor OR ‘guideline development group*’ OR ‘advisory group’ OR ‘knowledge user*’ OR knowledge-user* OR policy-mak* OR policymak* OR stakeholder* OR stake-holder* OR ‘stake holder*’ OR ‘end user*’ OR end-user*) | 382191 |
| **6** | **1 OR 2 OR 3 OR 4 OR 5** | 677737 |
| 7 | (MH "Professional Practice, Evidence-Based+") OR 'evidence based practice' OR ‘Literature Review as Topic’ OR ‘meta-analysis as topic’ OR ‘Systematic reviews as topic’ OR 'technology assessment' | 97824 |
| 8 | (knowledge N2 synthes*) OR (Health N2 Knowledge N2 Practice) | 1121 |
| 9 | (meta*) N2 (analysis OR regression OR review OR overview OR synthes*) | 99104 |
| 10 | meta-analy* OR meta-regression OR meta-review* OR meta-synthes* OR megasynthes* | 93732 |
| 11 | (evidence) N2 (synthes* OR summar*) | 10094 |
| 12 | (quantitative OR qualitative OR systematic OR rapid OR scoping OR realist OR Cochrane OR evidence) N2 (review* OR overview*) | 169935 |
| 13 | (HTA OR ‘health technology assessment’) | 18899 |
| **14** | **7 OR 8 OR 9 OR 10 OR 11 OR 12 OR 13** | 304529 |
| 15 | 'data visualization' OR 'implementation science' OR (information N1 dissemination) OR (health N2 information N2 communication) | 3599 |
| 16 | ‘summary of findings’ OR summary-of-findings OR table$ OR tabular | 33430 |
| 17 | ‘plain language summar*’ | 151 |
| 18 | TI (infographic* OR podcast* OR ‘visual abstract*’ OR ‘fact box*’ OR blogshot OR ‘blog shot’ OR podcast OR video OR ‘GRADE evidence profile’ OR ‘policy brief’ OR ‘league table*’ OR bulletin OR infogram OR brief OR briefing$ OR summary OR summaries OR graphic* OR audio) OR AB (infographic* OR podcast* OR ‘visual abstract*’ OR ‘fact box*’ OR blogshot OR ‘blog shot’ OR podcast OR video OR ‘GRADE evidence profile’ OR ‘policy brief’ OR ‘league table*’ OR bulletin OR infogram OR brief OR briefing$ OR summary OR summaries OR graphic* OR audio) | 203989 |
| 19 | (communicat* OR presentat*) N2 (finding*) | 2142 |
| **20** | **15 OR 16 OR 17 OR 18 OR 19** | 238202 |
| 21 | TI (perceive OR understand OR understanding OR acceptability OR effectiveness OR efficacy OR satisfaction OR usability) OR AB (perceive OR understand OR understanding OR acceptability OR effectiveness OR efficacy OR satisfaction OR usability) | 763180 |
| 22 | TI (usefulness OR credibility OR clarity OR comprehensive OR appeal OR appropriateness OR preference$) OR AB (usefulness OR credibility OR clarity OR comprehensive OR appeal OR appropriateness OR preference$) | 175329 |
| 23 | 21 OR 22 | 831235 |
| **24** | **6 AND 14 AND 20 AND 23** | **2327** |
|  | LIMIT 24 to academic journals | 2244 |

|  | **COCHRANE DATABASE OF SYSTEMATIC REVIEWS** |  |
| --- | --- | --- |
| 1 | 'administrative personnel' | 115 |
| 2 | ((health OR healthcare OR hospital*) NEAR/2 (administrator* OR analyst* OR decisionmak* OR decision-mak* OR manager* OR policymaker$ OR policy OR policies)) | 293 |
| 3 | (Decision NEAR/1 Making) OR (Policy NEAR/1 Making) | 14260 |
| 4 | ((decision$ OR policy OR policies) NEAR/2 (analysis OR maker$ OR making)) | 20326 |
| 5 | (analyst* OR clinician OR decision-mak* OR decisionmak* OR doctor OR ‘guideline development group*’ OR ‘advisory group’ OR ‘knowledge user*’ OR OR policymaker$ OR stakeholder$ OR ‘end user$’) | 15946 |
| **6** | **1 OR 2 OR 3 OR 4 OR 5** | 35144 |
| 7 | ('evidence based practice' OR ‘Literature Review as Topic’ OR ‘meta-analysis as topic’ OR ‘Systematic reviews as topic)’ | 38222 |
| 8 | (knowledge NEAR/1 synthesis) OR (knowledge NEAR/1 management) | 149 |
| 9 | (meta*) NEAR/2 (analysis OR regression OR review OR overview OR synthes*) | 22016 |
| 10 | meta-analy* OR meta-regression OR meta-review* OR meta-synthes* OR megasynthes* | 25457 |
| 11 | (evidence) NEAR/2 (synthes* OR summar*) | 742 |
| 12 | (quantitative OR qualitative OR systematic OR rapid OR scoping OR realist  OR evidence) NEAR/2 (review* OR overview*) | 13485 |
| 13 | (HTA OR ‘health technology assessment’) | 2231 |
| **14** | **7 OR 8 OR 9 OR 10 OR 11 OR 12 OR 13** | 48333 |
| 15 | 'data visualization' OR 'implementation science' OR (information N1 dissemination) | 2307 |
| 16 | ‘summary of findings’ | 13954 |
| 17 | ‘plain language summar*’ | 9613 |
| 18 | (infographic* OR podcast* OR ‘visual abstract*’ OR ‘fact box*’ OR blogshot OR ‘blog shot’ OR podcast OR video OR ‘GRADE evidence profile’ OR ‘policy brief’ OR ‘league table*’ OR bulletin OR infogram OR brief OR briefing$ OR summary OR summaries OR graphic* OR audio):ti,ab | 69917 |
| 19 | (communicat* OR presentat*) NEAR/2 (finding*) | 155 |
| **20** | **15 OR 16 OR 17 OR 18 OR 19** | 75523 |
| 21 | (perceive OR understand OR understanding OR acceptability OR effectiveness OR efficacy OR satisfaction OR usability) :ti,ab | 499060 |
| 22 | (usefulness OR credibility OR clarity OR comprehensive OR appeal OR appropriateness OR preference$):ti,ab | 40642 |
| 23 | 21 OR 22 | 52219 |
| **24** | **6 AND 14 AND 20 AND 23** | **2509** |
|  | Selected: 1777 reviews + 201 protocols. (Trials and other = 530) |  |

|  | **Web of Science, Science Citation Index, Social Science Citation Index** |  |
| --- | --- | --- |
| 1 | TS=('administrative personnel') | 1648 |
| 2 | TS=((health OR healthcare OR hospital$) NEAR/2 (administrator$ OR analyst$ OR decisionmaker$ OR manager$ OR policymaker$ OR policy OR policies OR provider)) | 114163 |
| 3 | TS=((Decision NEAR/1 Making) OR (Policy NEAR/1 Making)) | 368912 |
| 4 | TS=((decision$ OR policy OR policies) NEAR/2 (analys* OR analyz* OR maker$ OR making OR develop*)) | 527482 |
| 5 | TS= (analyst* OR clinician OR decisionmaker$ OR doctor OR ‘guideline development group*’ OR ‘advisory group’ OR ‘knowledge user$’ OR policy-maker$ OR stakeholder$ OR ‘stake holder*’ OR ‘end user$) | 537464 |
| **6** | **1 OR 2 OR 3 OR 4 OR 5** | 1055690 |
| 7 | TS=('evidence based practice' OR ‘Literature Review as Topic’ OR ‘meta-analysis as topic’ OR ‘Systematic reviews as topic’ ) | 98898 |
| 8 | TS=(knowledge NEAR/1 synthes*) | 1693 |
| 9 | TS=((meta*) NEAR/1 (analysis OR regression OR review OR overview OR synthes*)) | 287733 |
| 10 | TS=(meta-analy* OR meta-regression OR meta-review* OR meta-synthes* OR megasynthes*) | 224866 |
| 11 | TS=((evidence) NEAR/1 (synthes* OR summar*)) | 19177 |
| 12 | TS=((quantitative OR qualitative OR systematic OR rapid OR scoping OR realist OR Cochrane OR evidence) NEAR/1 (review* OR overview*)) | 262964 |
| 13 | TS=(HTA OR ‘health technology assessment’) | 17976 |
| **14** | **7 OR 8 OR 9 OR 10 OR 11 OR 12 OR 13** | 558459 |
| 15 | TS=('data visualization' OR 'implementation science' OR (information NEAR/1 dissemination) | 84360 |
| 16 | TS=(‘summary of findings’ OR summary-of-findings OR table$ OR tabular) | 252957 |
| 17 | TS=(‘plain language summar*’) | 7507 |
| 18 | TI=(infographic* OR podcast$ OR ‘visual abstract$’ OR ‘fact box*’ OR blogshot OR ‘blog shot’ OR podcast OR video OR ‘GRADE evidence profile’ OR ‘policy brief’ OR ‘league table*’ OR bulletin OR infogram OR brief OR briefing$ OR summary OR summaries OR graphic* OR audio) OR AB=(infographic* OR podcast* OR ‘visual abstract*’ OR ‘fact box*’ OR blogshot OR ‘blog shot’ OR podcast OR video OR ‘GRADE evidence profile’ OR ‘policy brief’ OR ‘league table*’ OR bulletin OR infogram OR brief OR briefing$ OR summary OR summaries OR graphic$ OR audio) | 816469 |
| 19 | TS=((communicat* OR presentat*) NEAR/1 (finding$)) | 4899 |
| **20** | **15 OR 16 OR 17 OR 18 OR 19** | 1079086 |
| 21 | TI=(perceive OR understand OR understanding OR acceptability OR effectiveness OR efficacy OR satisfaction OR usability) OR AB=(perceive OR understand OR understanding OR acceptability OR effectiveness OR efficacy OR satisfaction OR usability) | 4191736 |
| 22 | TI=(usefulness OR credibility OR clarity OR comprehensive OR appeal OR appropriateness OR preference$) OR AB=(usefulness OR credibility OR clarity OR comprehensive OR appeal OR appropriateness OR preference$) | 1033696 |
| 23 | 21 OR 22 | 5002679 |
| **24** | **6 AND 14 AND 20 AND 23** | **4637** |
|  | LIMIT 24 to journal articles | 4554 |

|  | **APA PSYCHINFO** |  |
| --- | --- | --- |
| 1 | 'administrative personnel' | 1239 |
| 2 | ((health OR healthcare OR hospital*) N2 (administrator* OR analyst* OR decisionmak* OR decision-mak* OR manager* OR official* OR policymak* OR policy-mak* OR policy OR policies OR provider)) | 239491 |
| 3 | DE "Decision Making" OR DE "Group Decision Making" OR DE "Management Decision Making" OR MM "Management Decision Making" OR MM "Policy Making" | 110660 |
| 4 | ((decision* OR policy OR policies) N2 (analys* OR analyz* OR maker* OR making OR develop*)) | 355355 |
| 5 | (analyst* OR clinician OR decision-mak* OR decisionmak* OR doctor OR ‘guideline development group*’ OR ‘advisory group’ OR ‘knowledge user*’ OR knowledge-user* OR policy-mak* OR policymak* OR stakeholder* OR stake-holder* OR ‘stake holder*’ OR ‘end user*’ OR end-user*) | 383013 |
| **6** | **1 OR 2 OR 3 OR 4 OR 5** | 618697 |
| 7 | MM "Evidence Based Practice" OR 'evidence based practice' OR ‘Literature Review as Topic’ OR ‘meta-analysis as topic’ OR ‘Systematic reviews as topic’ OR 'technology assessment' | 36237 |
| 8 | (knowledge N2 synthes*) OR (Health N2 Knowledge N2 Practice) | 27614 |
| 9 | (meta*) N2 (analysis OR regression OR review OR overview OR synthes*) | 43650 |
| 10 | meta-analy* OR meta-regression OR meta-review* OR meta-synthes* OR megasynthes* | 24460 |
| 11 | (evidence) N2 (synthes* OR summar*) | 5756 |
| 12 | (quantitative OR qualitative OR systematic OR rapid OR scoping OR realist OR Cochrane OR evidence) N2 (review* OR overview*) | 28785 |
| 13 | (HTA OR ‘health technology assessment’) | 1197 |
| **14** | **7 OR 8 OR 9 OR 10 OR 11 OR 12 OR 13** | 150125 |
| 15 | 'data visualization' OR 'implementation science' OR (information N1 dissemination) OR (health N2 information N2 communication) | 7387 |
| 16 | ‘summary of findings’ OR summary-of-findings OR table$ OR tabular | 30209 |
| 17 | ‘plain language summar*’ | 22 |
| 18 | TI (infographic* OR podcast* OR ‘visual abstract*’ OR ‘fact box*’ OR blogshot OR ‘blog shot’ OR podcast OR video OR ‘GRADE evidence profile’ OR ‘policy brief’ OR ‘league table*’ OR bulletin OR infogram OR brief OR briefing$ OR summary OR summaries OR graphic* OR audio) OR AB (infographic* OR podcast* OR ‘visual abstract*’ OR ‘fact box*’ OR blogshot OR ‘blog shot’ OR podcast OR video OR ‘GRADE evidence profile’ OR ‘policy brief’ OR ‘league table*’ OR bulletin OR infogram OR brief OR briefing$ OR summary OR summaries OR graphic* OR audio) | 257047 |
| 19 | (communicat* OR presentat*) N2 (finding*) | 2699 |
| **20** | **15 OR 16 OR 17 OR 18 OR 19** | 290029 |
| 21 | TI (perceive OR understand OR understanding OR acceptability OR effectiveness OR efficacy OR satisfaction OR usability) OR AB (perceive OR understand OR understanding OR acceptability OR effectiveness OR efficacy OR satisfaction OR usability) | 932899 |
| 22 | TI (usefulness OR credibility OR clarity OR comprehensive OR appeal OR appropriateness OR preference$) OR AB (usefulness OR credibility OR clarity OR comprehensive OR appeal OR appropriateness OR preference$) | 277285 |
| 23 | 21 OR 22 | 1139794 |
| **24** | **6 AND 14 AND 20 AND 23** | **5789** |
|  | LIMIT 24 to academic journals | 4648 |

|  | **SCOPUS** |  |
| --- | --- | --- |
| **1** | ( TITLE-ABS ( ( health OR healthcare OR hospital$ ) W/2 ( administrator$ OR analyst$ OR decisionmaker$ OR manager$ OR policymaker$ OR policy OR policies OR provider ) ) ) OR ( TITLE-ABS ( ( decision W/1 making ) OR ( policy W/1 making ) ) ) OR ( TITLE-ABS ( ( decision$ OR policy OR policies ) W/2 ( analys* OR analyz* OR maker$ OR making ) ) ) OR ( TITLE-ABS ( analyst* OR clinician OR decisionmaker$ OR ( guideline W/1 development W/1 group* ) OR ( advisory W/1 group ) OR ( knowledge W/1 user$ ) ' OR policymaker$ OR stakeholder$ OR ( end W/1 user$ ) ) ) | 894,656 |
| **2** | ( TITLE-ABS ( ( evidence W/1 based W/1 practice ) OR ( literature W/1 review W/2 topic ) OR ( meta-analysis W/1 topic ) OR ( systematic W/2 reviews W/2 topic' ) ) ) OR ( TITLE-ABS ( knowledge W/1 synthes* ) OR TITLE-ABS ( evidence W/1 synthesis ) OR TITLE-ABS ( evidence W/1 summar* ) ) OR ( TITLE-ABS ( ( meta* ) W/1 ( analysis OR regression OR review OR overview OR synthes* ) ) ) OR ( TITLE-ABS ( meta-analy* OR meta-regression OR meta-review* OR meta-synthes* OR megasynthes* ) ) OR ( TITLE-ABS ( ( quantitative OR qualitative OR systematic OR rapid OR scoping OR realist OR cochrane OR evidence ) W/1 ( review* OR overview* ) ) OR TITLE-ABS ( hta OR ( health W/1 technology W/1 assessment' ) ) ) | 554,202 |
| **3** | ( TITLE-ABS ( ( data W/1 visualization ) OR ( implementation W/1 science ) OR ( information W/1 dissemination ) ) ) OR ( TITLE-ABS ( summary W/2 findings ) OR TITLE-ABS ( plain W/2 language W/2 summar* ) OR TITLE-ABS ( ( communicat* OR presentat* ) W/1 ( finding$ ) ) ) OR ( TITLE-ABS ( infographic* OR podcast$ OR blogshot OR podcast OR video OR bulletin OR infogram OR brief OR briefing$ OR summary OR summaries OR graphic* OR audio ) ) | 1,916,344 |
| **4** | TITLE-ABS ( perceive OR understand OR understanding OR acceptability OR effectiveness OR efficacy OR satisfaction OR usability ) OR TITLE-ABS ( usefulness OR credibility OR clarity OR comprehensive OR appeal OR appropriateness OR preference ) | 7,418,995 |
| **5** | 1 AND 2 AND 3 AND 4 | 1328 |
|  | LIMIT 5 TO JOURNAL ARTICLES | 1236 |
|  |  |  |

END
