# Supplementary material for: The effectiveness and acceptability of evidence synthesis summary formats for clinical guideline development groups: a mixed-methods systematic review
Source: Implement Sci. 2022 Oct 27;17:74. doi: 10.1186/s13012-022-01243-2 (PMC9615384; doi:10.1186/s13012-022-01243-2)
Supplement: Supplementary file 4 — Additional file 4. Quantitative findings. [file 13012_2022_1243_MOESM4_ESM.docx]

# **Appendix 4.** Quantitative findings

*Items in blue were main results reported (in abstract).*

*Items which are double asterisked** are the quantitative findings which converge with qualitative findings/recommendations.*

Buljan 2018

*Understanding/Knowledge*

1. There were no significant differences between the infographic and PLS groups in the knowledge test score (Table 2).
2. In the overall sample, the participants who received the infographic had a similar knowledge score to those reading the PLS (Table 3).
3. No difference in knowledge between consumers given infographic 7(6-7) vs. PLS vs. 7(6-7) p=.511
4. A significant predictor of the knowledge score was awareness about Cochrane systematic reviews (OR 5 5.33; 95% CI: 1.71e16.62), explaining 13.4% of the variance.**
5. There were no significant differences among the three groups in the knowledge score (Table 2).
6. None of the demographic characteristics or secondary outcomes significantly predicted the knowledge test score.
7. No difference between consumers given PLS and doctors given PLS in knowledge (7, 6-7 vs. 8, 7-9)
8. No difference between consumers given Infographic and doctors given infographic in knowledge 7(6-7) vs. 8(6-8)
9. Significant predictors were health numeracy (OR 5 1.48; 95% CI: 1.25e1.74) and reading experience (OR 5 1.06; 95% CI: 1.04e1.09), explaining 17.0% of the variance of the knowledge score. **

*User-friendliness*

1. Infographic consumers user-friendliness 30(25.5-34.5) vs. infographics doctors user-friendliness 36(30.9-40.0)
2. Difference in user-friendliness between consumers given infographic vs. PLS 30(25.5-34.5) vs. 21(19-25) p<.001
3. Infographic users scored higher on user-friendliness than those reading the PLS (Table 3) **
4. In the PLS group, consumers reported lower satisfaction with that format, compared with ~~students and~~ doctors (Table 2). User-friendliness: 21.0(19-25) vs. doctors 29(26.8-36.2)

*Reading experience*

1. However, the group that read the SA had significantly lower scores on the measures of reading experience and user-friendliness of the material (Table 2).
2. In the PLS group, consumers reported lower satisfaction with that format, compared with ~~students and~~ doctors (Table 2). Reading experience: 22.5(19.0-27.4) vs. doctors 32.0(30.0-39.9) p<.001
3. Consumers infographic reading experience 33(28-36) vs. doctors infographics reading experience 37(26.8-41.3)
4. Difference in reading experience between consumers given infographic 33(28-36) vs. PLS 22.5(19-27.4) p<.001
5. However, infographic users scored higher on reading experience (33, 28-36 vs. 22.5, 19-27.4) **

**Carasco-Labra**

*Understanding*

1. Participants allocated to the new format consistently had a higher proportion of correct answers compared to those who were allocated to the current format (difference in proportions between groups ranging from 0% to 63%) (see Table 5).
2. No difference between formats: Ability to interpret footnotes For the outcome adverse events, why is the quality of evidence rated as low? Risk difference: 7%(-2 to 15) p value: 0.18
3. No difference between formats: Ability to interpret risk Will fewer children <5 years old have diarrhoea if they take the probiotics? 96 96 Risk difference: 0% (-5.3 to 5.4) p value: 0.99
4. New SoF format better than current: Ability to determine risk difference How many fewer children <5 years will have diarrhoea if they have probiotics than if they do not? 98 35 Risk difference: 63% (54.6 to 71) p value: <0.001
5. New SoF format better than current: Understanding of quality of evidence and treatment effect Which of the following statements best represents the results informing the outcome adverse events? 88 26 Risk difference: 62% (52 to 71) p value: <0.001
6. No difference between formats: Understanding of quality of evidence In children <5 years old, what result is most certain? 97 90 Risk difference: 7% (0.1 to 12.4) p value: 0.06
7. No difference between formats: Ability to relate N of participant/studies and outcomes How many participants and studies are informing the outcome adverse events? 95 98 Risk difference: -3% (-7.5 to 1.7) p value: 1.00
8. No difference between formats: Ability to quantify risk In childrenO5 years old, how many fewer or more children will have diarrhoea if they took probiotics as an adjunct to antibiotics compared to those who did not take probiotics? 94 88 Risk difference: 6% (0.1 to 13.3) p value: 0.06
9. For (understanding) question 1, (ability to interpret footnotes) only years of experience modified the estimate by more than 10% [adjusted odds ratio (OR): 1.83; 95% CI (0.91, 3.67); P-value 5 0.088].
10. For (understanding) question 2 (ability to interpret risk), years of experience, familiarity with GRADE, and level of training modified the outcome by more than 10% [adjusted OR: 0.72; 95% CI (0.20, 2.56); P-value 5 0.6], but these modifications were not significant.
11. For the remaining five (understanding) questions, there were no covariates modifying the outcome.

*Accessibility of information*

1. Participants allocated to the new format considered, on average, that the information was more accessible across all domains assessed compared to the current formats (see Table 6). The overall accessibility assessment per domain also favored the new format (MD 0.3; SE 0.11; Pvalue <0.001).
2. It was easy to find the information about the effects. The adjusted analysis for the statement ‘‘It was easy to find the information about the effects’’ [MD 0.4; standard error (SE) 0.19; P-value 5 0.04] and
3. ‘It was easy to understand the information?’’ (MD 0.5; SE 0.20; P-value 5 0.017) showed a nonstatistically significant difference between the two groups (P-value adjusted for multiple comparisons).
4. Participants allocated to the new format considered that these items displayed review results in a way that was more helpful for decision making than the current ones (MD 0.5; SE 0.18; P-value 5 0.011). ‘The information is presented in a way that would help me making a decision’

*Satisfaction*

1. More than 72% (203) would like to see the definition of each category for the quality of the evidence within the SoF table**
2. 60% (171) think that the ‘‘number of participants/studies’’ column can be eliminated and the information can be accommodated in the ‘‘outcome’’ column
3. 63% (178) mentioned that the ‘‘comments’’ column is not necessary,
4. 86% (243) would like to see the reasons for downgrading the quality of evidence within the table, **
5. 88% (251) favored the inclusion of the ‘‘what happens’’ column
6. 88% (250) considered that an additional column showing the risk and MDs along with their 95% CIs should be included (see Table 7).

*Preference*

1. Participants in both groups consistently preferred the new to the current format (mean/SD new format shown first 2.9/1.6; mean/SD current SoF table format shown first 2.8/1.7). The adjusted analysis also suggested a preference for the new over the current format. However, there were no statistically significant differences between the two groups for this outcome in either analysis.
2. Overall, participants preferred the alternative to the current formats (MD/SD: 2.8/1.6).

**Opiyo**

*Understanding*

1. There were no significant differences between packs in the odds of correct responses (adjusted ORs: pack B versus A 0.59, 95% CI 0.32 to 1.07; pack C versus A 0.66, 95% CI 0.36 to 1.21; table 3).
2. There was some evidence of differences in the odds of correct responses across different groups of healthcare professionals (p=0.057).
3. Results of sub-group analyses (although not statistically significant) suggested that both pack B (systematic reviews with SoF tables) and pack C (the three component, graded entry pack) improved understanding for policymakers (pack B: OR 1.5, 95% CI 0.15 to 15.15; pack C: OR 1.5, 95% CI 0.64 to 3.54) and trainee paediatricians (pack B: OR 1.3, 95% CI 0.37 to 4.66; pack C: OR 1.78, 95% CI 0.43 to 7.33).

*Clarity and accessibility (composite)*

1. ‘Graded-entry’ formats (pack C), compared to systematic review formats (pack A), were associated with a significantly higher mean ‘value and accessibility’ score (adjusted mean difference 0.52, 95% CI: 0.06 to 0.99; table 3). [value and accessibility = clarity in abstract] **
2. Similarly, pack C, compared to pack A, was associated with a 1.5 higher odds of judgments about the quality of evidence for critical neonatal outcomes being clear and accessible (adjusted OR: 1.52, 95% CI 1.06 to 2.20; table 3).
3. There was no evidence that pack B (systematic reviews with SoF tables) improved this composite score compared to pack A (adjusted mean difference: 20.11, 95% CI 20.71 to 0.48; table 3).
4. More than half of the respondents (60%) found systematic reviews to be more difficult to read compared to narrative reports, but some (17%) responded that systematic reviews were easy to read. **
5. About half of the participants (51%) found systematic reviews to be easier to read compared to summary-of-findings tables (26%).

(Preference)

1. A higher proportion of participants preferred evidence summarised in narrative report formats to the full version of the systematic reviews (53% versus 25%).
2. 40% of participants strongly preferred the SoF table to the systematic review (32%) but 29% were neutral. **
3. 38% strongly preferred the narrative report vs. 23% that strongly preferred the SoF table. 39% were neutral. **

**Rosenbaum**

RCT1:

*User satisfaction*

*Perceived understanding and ease of use*

1. No difference between with and without SoF table: Review authors have indicated what they believe are the most important outcomes p = .93
2. No difference between with and without SoF table: It was easy to find this information (about which outcomes are important) p=.29
3. 68% (with sof table) vs. 40% (without sof table) It was easy to find results for important outcomes p=.021**
4. No difference between with and without SoF table: It was easy to find quality of the evidence for important outcomes
5. No difference between with and without SoF table: It was easy to understand the main findings. = .31
6. No difference between with and without SoF table: Main findings are presented in a such a way that they would be helpful to me in making a decision. P=.54
7. 41 (with sof table) vs. 17% (without sof table) Overall perceived accessibility p = .037**
8. Although most people felt the results were accessible with or without the Summary of Findings tables, many people gave the wrong answer when they were asked to calculate the intervention group risk in a structured discussion following the trial
9. Presenting results of the intervention group risk framed as an absolute difference (9 fewer per 1,000) caused comprehension problems or uncertainty.
10. Eighty-one percent agreed or strongly agreed that Cochrane reviews should include SoF tables, 65% with the proposed format**
11. 75% found the explanation sheet helpful.**

RCT2:

*Understanding*

1. There were large differences in the proportion that answered correctly questions about the risk in the control group (44 versus 93%, p=0.003) and the risk in the intervention group (11 versus 87%, p<0.001).
2. No difference in correct understanding between without SoF and with SoF: how much confidence to review author shave in estimated effect of stockings on the risk of symptom-less DVT? 67 vs. 87%. P = .18
3. No difference of correct understanding (all 6 correctly) between without SoF and with SoF: what are the most important outcomes? 33 vs. 53% p = .27

*Time spent finding key results*

1. There were also large differences in actual time spent as well as the proportion that agreed or strongly agreed that it was easy to find information about the quality of evidence for the main outcomes (24 versus 73%, p=0.005, Pearsons Chi-square).
2. Difference in mean time spent finding answer between without sof table and with SoF table groups: 4 min vs 1.5. p = .002**
3. No difference in mean time spent finding answer between without sof table and with SoF table groups: how much confidence to review author shave in estimated effect of stockings on the risk of symptom-less DVT? 1.5 vs. 2.1 min p = .47
4. No difference in mean time spent finding answer between without sof table and with SoF table groups: what are the most important outcomes? 1.9 vs. 2.0 min p = .88
5. No difference in mean time spent finding answer between without sof table and with SoF table groups: what would be the risk of they wore stockings: 2.8 vs. 1.3 min. p = .118
6. Most participants were positive about including the SoF tables and the format: 88% agreed or strongly agreed that Cochrane reviews should have Summary of Findings tables, 84% with the format proposed, **
7. 77% that the explanations were helpful. **
8. Most (67%) preferred placing Summary of Findings tables after the abstract.
