# Supplementary material for: The effectiveness and acceptability of evidence synthesis summary formats for clinical guideline development groups: a mixed-methods systematic review
Source: Implement Sci. 2022 Oct 27;17:74. doi: 10.1186/s13012-022-01243-2 (PMC9615384; doi:10.1186/s13012-022-01243-2)
Supplement: Supplementary file 5 — Additional file 5. Qualitative synthesis recommendations. [file 13012_2022_1243_MOESM5_ESM.docx]

**Appendix 5.** Qualitative Synthesis Recommendations

| **Key**  * Supported by both qualitative and quantitative evidence  Specifically expressed by clinicians^a^, policy/decision makers^b^, healthcare managers^c^, content experts/academics^d^, guideline committees^e,^ patient representatives^f^  *For studies reporting on the same trial (i.e., Hartling 2016 and Hartling 2018* [1] *and Smith 2019 and Totten 2019* [2]*, only one paper is cited as to not visually misrepresent recommendations as being supported by more studies than it is)* |
| --- |

| **Presenting Information** |
| --- |
| **All reviews**  **Content**  First page   1. List authors [3,4] 2. Give publication date [3–5] 3. Detail key messages [1,3,4,6,7] separated by subheadings [3] 4. Describe relevance for practice/policies [4]^b^   General   1. Plain language [1,3,6,8–10] and jargon-free [5] 2. Avoid repetition [3,8,11] 3. Avoid abbreviations [6]^b^; if necessary, don’t define in footnotes [1]^b^ 4. Consider framing title as question [6]^b^ 5. Prominent subheadings [5] 6. Provide background [3] 7. Give PICOS information [8,10,12] and characteristics of included studies [13] 8. Report what worked and what didn’t [5] 9. Use ‘explanations’ or ‘clarifications’ instead of ‘footnotes’ [11] 10. If no information is available, clearly indicate that [8,14] 11. Rank evidence and recommendations [1,5,8] 12. Don’t put implications in summary tables [11]; keep separate from review [8] ^a^ 13. Noticeable hyperlinks to supporting documents (full review, data, individual studies, etc.) [1–3,3,5,15] 14. Clear referencing style to not confuse with numeric data[12] 15. Consistency between numbers in tables/text [6]^b^ 16. Avoid statistical information [3]^b^ 17. Succinct methods, data sources, review approach, and criteria [1]^b^   **Structure**   1. Concise [3,5,8,9,11,16] 2. Brief narrative report [4,7]* 3. Structured [1,4,5,7,9]* 4. Intuitive presentation [8]^a^ 5. High-level one page summary [3,15] 6. Consistent presentation [9] 7. Use Summary of findings (SoF) tables [3,6–8,11,13,17]*, allowing for qualitative data [11] in prominent positions [12]   **Typography**   1. Use bullet points [3,8,18] 2. Flag important information [8,11] by bolding/highlighting [8]^a^, don’t put in footnotes[11] 3. Use greyscale-friendly colours [1]^b^ 4. Avoid dense information. Promote white space [1,3,4]   **Results, tables & figures**   1. Use variations in cell colour/fonts for multiple control group risks [12] 2. Shade rows [1]^b^ 3. Decrease numeric/statistical data [3,9,12] 4. Use absolute numbers, not probabilities [11] 5. Present numbers in tables and/or visually [9]; use icons [3] or graphics [4]^b^[19] 6. Limit visual information to single table/image [9] 7. Balance visual and textual information [1]^b^ 8. Use multiple columns [3] 9. Don’t break tables over multiple pages [3,6,10] 10. Avoid forest plots [3] 11. Keep footnotes on same page as tables [11]   **Diagnostic Test Accuracy (DTA) Specific** [11]  **Content**  44. Put information about index/reference tests, sensitivity/specificity, and multiple prevalence estimates in column headers  45. Present data for two tests in one table (for easy comparison)  **Network Meta-Analysis (NMA) Specific [16] Structure**  46. Put question at the top  47. SUCRA format  48. Display information by outcome  **Content**  49. One outcome per SoF table  50. SoF table with absolute/relative effects, and certainty of evidence  51. Include number of RCTs in direct estimates  52. Detail number of participants in specific pairwise comparisons  53. Include SoF footnotes if necessary  **Results, tables & figures**  54. Present NMA relative and absolute effects without direct and indirect estimates  55. Provide credible intervals  56. Rank information  57. Rank evidence  58. Clearly define column labels  59. Provide network geometry  **Typography**  60. Be judicious in use of colours  61. Consider those with colour visual impairment  **Updating Reviews [18] Content**  62. Narrative summaries preferred over quantitative tables  **Typography**  63. Avoid tracked changes mode (i.e., in MS Word)  64. Use colour coding, bolding, or different fonts for information that changed |
| **Tailoring Information** |
| **All reviews**  **Structure**   1. Standard formatting to aid familiarity with repeated exposure [5] 2. Flexibility in delivery (electronic/PDF, printable, not requiring internet) [5] 3. Easily extractable information to forward to colleagues and use personally [3,4] 4. Structure question around condition first, then intervention [2]^a^ 5. Present positive results first, then negative [11]^a^ 6. AMRaD format [8]^a^ 7. Avoid academic formatting [3] ^b^ 8. No more than 1 page [6]^c^ 9. No more than 3 pages [1]^b^ 10. Visual format might be more useful [2] ^e^   **Content**   1. Clarify audience [1]^b^ 2. Accommodate different learning styles [2] 3. Choice and control over the amount of detail received [2,3,5] 4. Consider interpretation aids for statistics [2] ^a^ 5. Short summary with conclusions for key questions most helpful [18] ^a^ 6. Use end user’s native language [4]^b^ 7. Title, key messages, link to more detail [4]^b^ 8. Don’t include methodology information [9]^f^ [3]^b^ 9. Provide inclusion and exclusion criteria [2]^d^   **Network Meta-Analysis (NMA) Specific [16]  Structure**   1. Present SoF tables such that they can be used as appendixes in clinical guidelines   **Updating Reviews [18] Content**   1. Those involved with setting guidelines may want to see both old and new information 2. Want to see what information changed^b,d^ 3. Prefer only the most up to date version^a^ 4. Only need short summary on conclusion; citations not necessary^a^ |
| **Contextualising Findings** |
| **All reviews**  **Content**   1. Legal/political conditions in country/region [4]^b^ 2. Framed within local [2,5,6], national, [5,9] or broader context [6]^b^ 3. Implementation/application information [3,8,9] 4. Cost analyses [5,6] 5. Limitations of findings [13] 6. Ramifications of methodological approaches [13] 7. Recommendations for practice/policies [2,3,5,6,12] and future research needs [13] 8. Clinical scenario example [6,13] and bottom line [8]^a^ 9. Effective intervention details [3,12] to help implementation, (e.g., dosages [3,8], trade names [8]^a^, treatment duration/frequency [3,8], costs [8]^a^, settings [3,15], evaluators of treatments [8]^a^, prevalence estimates [3,11], population characteristics [2,3,8,15] 10. Not interested in interventions with no effects or search results information [3] ^b^   **Updating Reviews [18]  Content**   1. Show how new findings changed conclusions   **Network Meta-Analysis (NMA) Specific** [10]  **Content**   1. Provide interpretations of findings 2. Detail reference comparator interventions |
| **Quality of Evidence** |
| **Content**   1. Include quality assessment of evidence/study quality [1,2,9,10,13,20]* 2. Provide distinct explanations of rating scale (GRADE) [7–11,14,17,20]* 3. Detail how authors arrived at assessments of quality [4,7,20]* in footnotes [11] 4. Rank or group studies [1,8] 5. Appreciate methodology details and limitations [3]^c^ |
| **Trust in Producers and Summary** |
| **Content**   1. Include conflict of interest statements (of primary studies) [4]^b^ and summary producers [8] ^a^ 2. Include funding sources [3] 3. Include authors’ names [5,6,8,15] 4. Put logos on first page [1,4–6] 5. Include clear references [6]^b^ 6. Establish credibility of research evidence [5] |
| **Knowledge Required** |
| **Content**   1. Avoid field-specific or technical jargon (e.g., ‘scaling up’, ‘EBM’, ‘PICO’) [6,7,15] 2. Avoid abbreviations (e.g., RR for relative risk, CI for confidence intervals) [12] 3. Provide information on nature of systematic review and standard steps [6,13,15,19]*   **Results, tables & figures**   1. Define ‘no data available’ and reasons for empty cells [12] 2. Define statistical terms [3,4,6,11,12,15,17]* 3. Define relative risk and confidence interval in forest plots [3] 4. Forest plots are difficult to understand [3] 5. Provide interpretation of statistical results [7,17,19]* 6. Define column labels [11] 7. Avoid probabilities [9,11] 8. Use similar table formats to aid readability with repeated exposure makes tables easier to read [12]   **Network Meta-Analysis (NMA) Specific [16]**  **Content**   1. Define NMA specific terminology (in footnotes) 2. Describe ranking system 3. Describe use of multiple baseline risks in context of NMA |

**References**

1. Hartling L, Gates A, Pillay J, Nuspl M, Newton AS. Development and Usability Testing of EPC Evidence Review Dissemination Summaries for Health Systems Decisionmakers. Agency for Healthcare Research and Quality (US); 2018; Available from: http://ovidsp.ovid.com/ovidweb.cgi?T=JS&PAGE=reference&D=medp&NEWS=N&AN=30507111

2. Totten AM, Smith C, Dunham K, Jungbauer RM, Graham E. Improving Access to and Usability of Systematic Review Data for Health Systems Guidelines Development. Agency for Healthcare Research and Quality (US); 2019; Available from: http://ovidsp.ovid.com/ovidweb.cgi?T=JS&PAGE=reference&D=medp&NEWS=N&AN=31013017

3. Marquez CJ Alekhya Mascarenhas; Jassemi, Sabrina; Park, Jamie; Moore, Julia E; Blaine, Caroline; Bourdon, Gertrude; Chignell, Mark; Ellen, Moriah E; Fortin, Jacques; Graham, Ian D; Hayes, Anne; Hamid, Jemila S; Hemmelgarn, Brenda R; Hillmer, Michael P; Holmes, Bev; Holroyd-Leduc, Jayna; Hubert, Linda; Hutton, Brian; Kastner, Monika; Lavis, John N; Michell, Karen; Moher, David; Ouimet, Mathieu; Perrier, Laure; Proctor, Andrea; Noseworthy, Tom; Schuckel, Victoria; Stayberg, Sharlene; Tonelli, Marcello; Tricco, Andrea C; Straus, Sharon E. Enhancing the uptake of systematic reviews of effects: what is the best format for health care managers and policy-makers? A mixed-methods study. 2018;13:84-NA.

4. Busert LK, Mütsch M, Kien C, Flatz A, Griebler U, Wildner M, et al. Facilitating evidence uptake: Development and user testing of a systematic review summary format to inform public health decision-making in German-speaking countries. Health Research Policy and Systems [Internet]. 2018;16. Available from: https://www.scopus.com/inward/record.uri?eid=2-s2.0-85049782278&doi=10.1186%2fs12961-018-0307-z&partnerID=40&md5=8a60b2081f09fd2655dac0ddecb23467

5. Dobbins MT Helen; O’Brien, Mary Ann; Duggan, Melissa. Use of systematic reviews in the development of new provincial public health policies in Ontario. 2004;20:399–404.

6. Rosenbaum SE, Glenton C, Wiysonge CS, Abalos E, Mignini L, Young T, et al. Evidence summaries tailored to health policy-makers in low- and middle-income countries. Bull World Health Organ. 2011;89:54–61.

7. Opiyo N, Shepperd S, Musila N, Allen E, Nyamai R, Fretheim A, et al. Comparison of Alternative Evidence Summary and Presentation Formats in Clinical Guideline Development: A Mixed-Method Study. PLoS ONE [Internet]. 2013;8. Available from: ://WOS:000315210400056

8. Perrier LK M Ryan; Straus, Sharon E. An iterative evaluation of two shortened systematic review formats for clinicians: a focus group study. 2014;21:e341-6.

9. Buljan I, Tokalić R, Roguljić M, Zakarija-Grković I, Vrdoljak D, Milić P, et al. Comparison of blogshots with plain language summaries of Cochrane systematic reviews: a qualitative study and randomized trial. Trials. 2020;21:426.

10. Yepes-Nunez JJ, Li SA, Guyatt G, Jack SM, Brozek JL, Beyene J, et al. Development of the summary of findings table for network meta-analysis. Journal of Clinical Epidemiology. 2019;115:1–13.

11. Mustafa R, Wiercioch W, Brozek J, Lelgemann M, Buehler D, Garg A, et al. Enhancing the acceptance and implementation of grade summary tables for evidence about diagnostic tests. BMJ Quality and Safety. 2013;22:A36.

12. Rosenbaum SG Claire; Nylund, Hilde Kari; Oxman, Andrew D. User testing and stakeholder feedback contributed to the development of understandable and useful Summary of Findings tables for Cochrane reviews. 2010;63:607–19.

13. Hartling L, Guise JM, Hempel S, Featherstone R, Mitchell MD, Motu’apuaka ML, et al. Fit for purpose: Perspectives on rapid reviews from end-user interviews. Systematic Reviews [Internet]. 2017;6. Available from: https://www.scopus.com/inward/record.uri?eid=2-s2.0-85013130994&doi=10.1186%2fs13643-017-0425-7&partnerID=40&md5=d0ea651ce13e9b75e702f2b5a9e822cc

14. Perrier LK M Ryan; Straus, Sharon E. A usability study of two formats of a shortened systematic review for clinicians. 2014;4:e005919-NA.

15. Steele R. Mental health clinicians views of summary and systematic review utility in evidence-based practice. Health Information and Libraries Journal [Internet]. Available from: ://WOS:000627057300001

16. Babatunde OO, Tan V, Jordan JL, Dziedzic K, Chew-Graham CA, Jinks C, et al. Evidence flowers: An innovative, visual method of presenting “best evidence” summaries to health professional and lay audiences. Research Synthesis Methods. 2018;9:273–84.

17. Rosenbaum SG Claire; Oxman, Andrew D. Summary-of-findings tables in Cochrane reviews improved understanding and rapid retrieval of key information. 2010;63:620–6.

18. Newberry SJ, Shekelle PG, Vaiana M, Motala A. Reporting the Findings of Updated Systematic Reviews of Comparative Effectiveness: How Do Users Want To View New Information? Agency for Healthcare Research and Quality (US); 2013; Available from: http://ovidsp.ovid.com/ovidweb.cgi?T=JS&PAGE=reference&D=medp&NEWS=N&AN=23785728

19. Buljan I, Malički M, Wager E, Puljak L, Hren D, Kellie F, et al. No difference in knowledge obtained from infographic or plain language summary of a Cochrane systematic review: three randomized controlled trials. J Clin Epidemiol. 2018;97:86–94.

20. Carrasco-Labra A, Brignardello-Petersen R, Santesso N, Neumann I, Mustafa RA, Mbuagbaw L, et al. Improving GRADE evidence tables part 1: a randomized trial shows improved understanding of content in summary of findings tables with a new format. Journal of Clinical Epidemiology. Elsevier; 2016;74:7–18.
