# Supplementary material for: The effectiveness and acceptability of evidence synthesis summary formats for clinical guideline development groups: a mixed-methods systematic review
Source: Implement Sci. 2022 Oct 27;17:74. doi: 10.1186/s13012-022-01243-2 (PMC9615384; doi:10.1186/s13012-022-01243-2)
Supplement: Supplementary file 6 — Additional file 6: Figures 6, 7, and 8. Recommendations for Practice. [file 13012_2022_1243_MOESM6_ESM.pptx]

## Slide 1
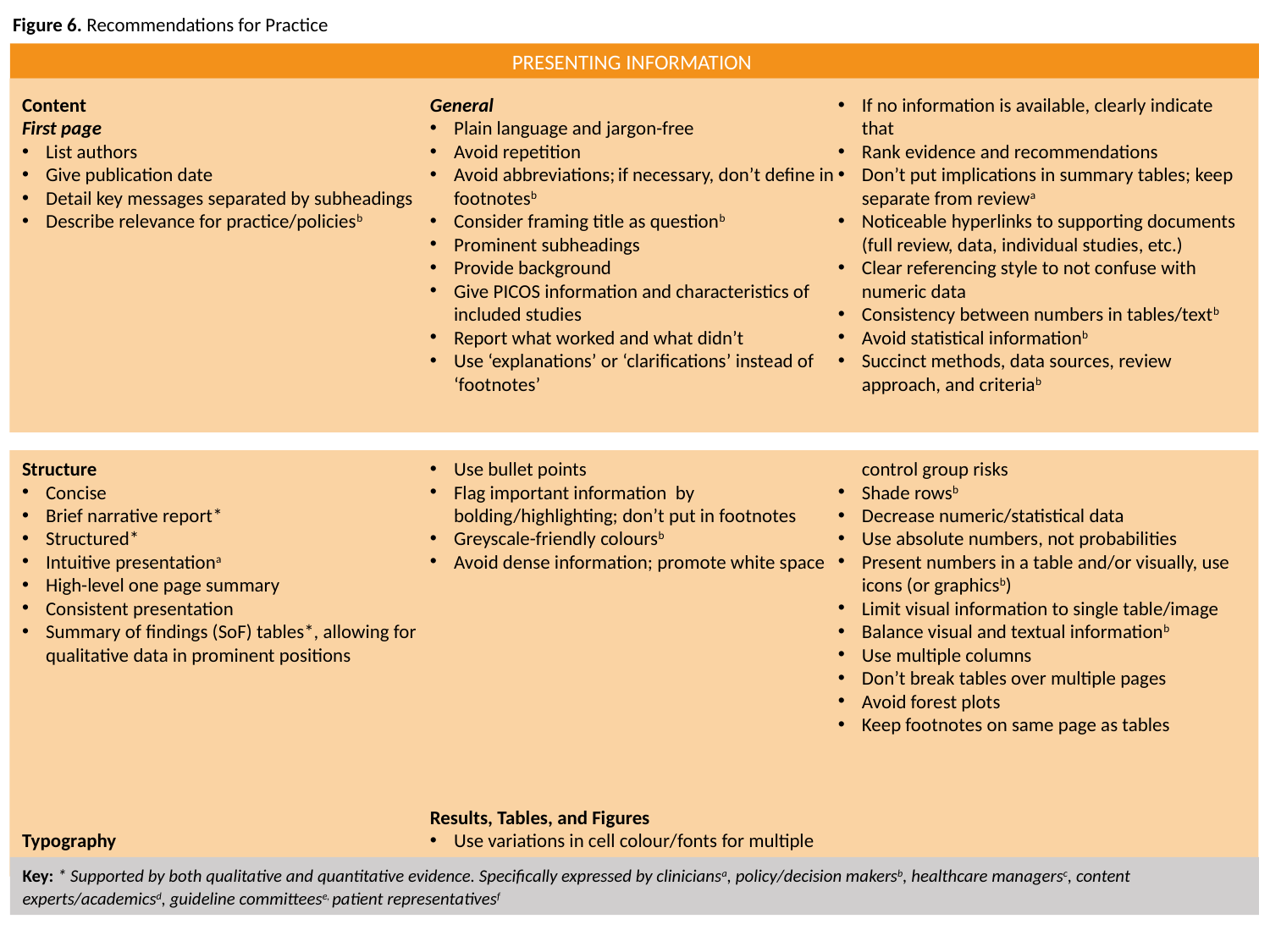

Figure 6. Recommendations for Practice
PRESENTING INFORMATION
Content
First page
List authors
Give publication date
Detail key messages separated by subheadings
Describe relevance for practice/policiesb
General
Plain language and jargon-free
Avoid repetition
Avoid abbreviations; if necessary, don’t define in footnotesb
Consider framing title as questionb
Prominent subheadings
Provide background
Give PICOS information and characteristics of included studies
Report what worked and what didn’t
Use ‘explanations’ or ‘clarifications’ instead of ‘footnotes’
If no information is available, clearly indicate that
Rank evidence and recommendations
Don’t put implications in summary tables; keep separate from reviewa
Noticeable hyperlinks to supporting documents (full review, data, individual studies, etc.)
Clear referencing style to not confuse with numeric data
Consistency between numbers in tables/textb
Avoid statistical informationb
Succinct methods, data sources, review approach, and criteriab
Structure
Concise
Brief narrative report*
Structured*
Intuitive presentationa
High-level one page summary
Consistent presentation
Summary of findings (SoF) tables*, allowing for qualitative data in prominent positions
Typography
Use bullet points
Flag important information by bolding/highlighting; don’t put in footnotes
Greyscale-friendly coloursb
Avoid dense information; promote white space
Results, Tables, and Figures
Use variations in cell colour/fonts for multiple control group risks
Shade rowsb
Decrease numeric/statistical data
Use absolute numbers, not probabilities
Present numbers in a table and/or visually, use icons (or graphicsb)
Limit visual information to single table/image
Balance visual and textual informationb
Use multiple columns
Don’t break tables over multiple pages
Avoid forest plots
Keep footnotes on same page as tables
Key: * Supported by both qualitative and quantitative evidence. Specifically expressed by cliniciansa, policy/decision makersb, healthcare managersc, content experts/academicsd, guideline committeese, patient representativesf

## Slide 2
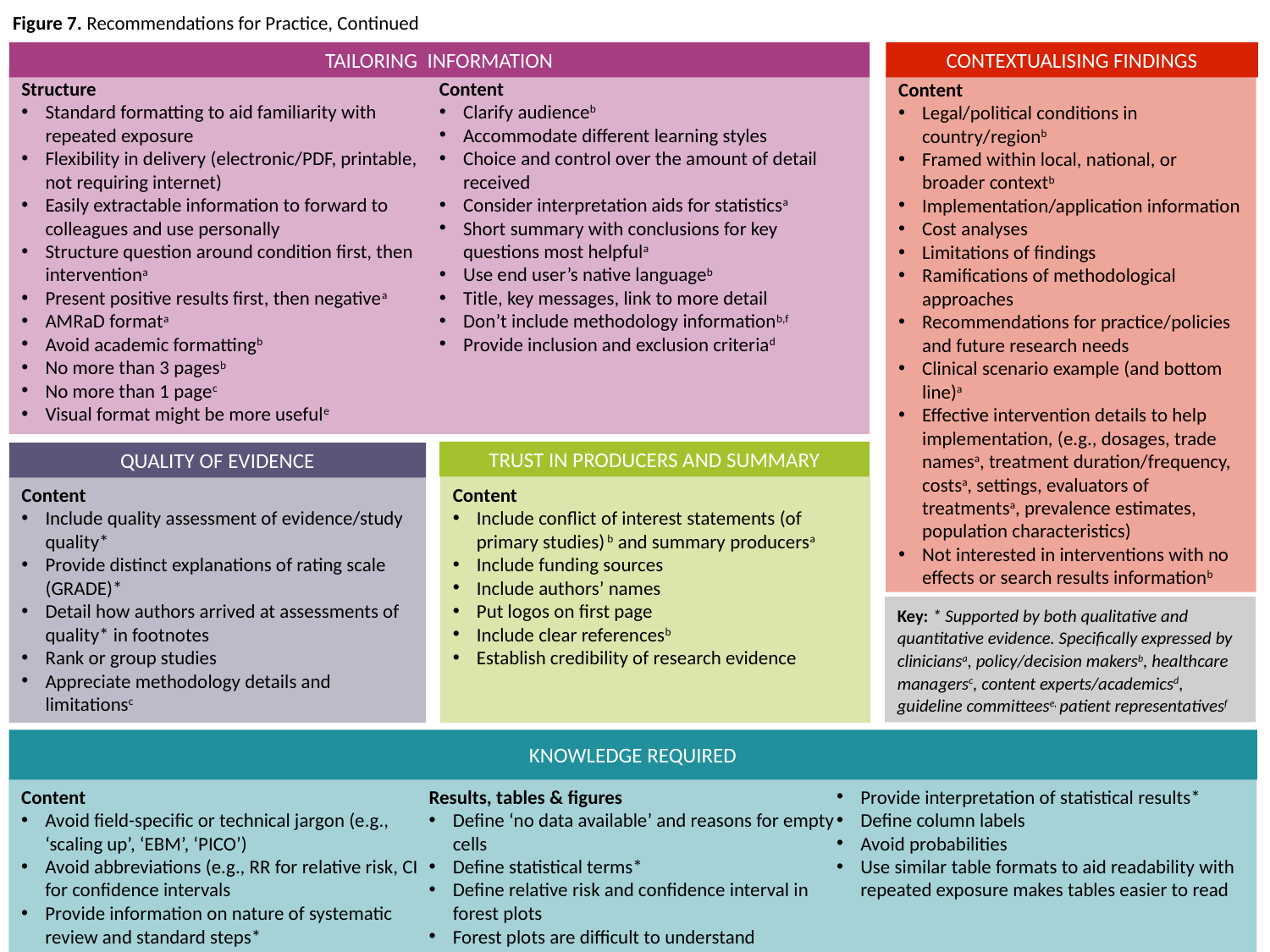

TAILORING INFORMATION
CONTEXTUALISING FINDINGS
Figure 7. Recommendations for Practice, Continued
Structure
Standard formatting to aid familiarity with repeated exposure
Flexibility in delivery (electronic/PDF, printable, not requiring internet)
Easily extractable information to forward to colleagues and use personally
Structure question around condition first, then interventiona
Present positive results first, then negativea
AMRaD formata
Avoid academic formattingb
No more than 3 pagesb
No more than 1 pagec
Visual format might be more usefule
Content
Clarify audienceb
Accommodate different learning styles
Choice and control over the amount of detail received
Consider interpretation aids for statisticsa
Short summary with conclusions for key questions most helpfula
Use end user’s native languageb
Title, key messages, link to more detail
Don’t include methodology informationb,f
Provide inclusion and exclusion criteriad
Content
Legal/political conditions in country/regionb
Framed within local, national, or broader contextb
Implementation/application information
Cost analyses
Limitations of findings
Ramifications of methodological approaches
Recommendations for practice/policies and future research needs
Clinical scenario example (and bottom line)a
Effective intervention details to help implementation, (e.g., dosages, trade namesa, treatment duration/frequency, costsa, settings, evaluators of treatmentsa, prevalence estimates, population characteristics)
Not interested in interventions with no effects or search results informationb
KNOWLEDGE REQUIRED
TRUST IN PRODUCERS AND SUMMARY
QUALITY OF EVIDENCE
Content
Include quality assessment of evidence/study quality*
Provide distinct explanations of rating scale (GRADE)*
Detail how authors arrived at assessments of quality* in footnotes
Rank or group studies
Appreciate methodology details and limitationsc
Content
Include conflict of interest statements (of primary studies) b and summary producersa
Include funding sources
Include authors’ names
Put logos on first page
Include clear referencesb
Establish credibility of research evidence
Key: * Supported by both qualitative and quantitative evidence. Specifically expressed by cliniciansa, policy/decision makersb, healthcare managersc, content experts/academicsd, guideline committeese, patient representativesf
Content
Avoid field-specific or technical jargon (e.g., ‘scaling up’, ‘EBM’, ‘PICO’)
Avoid abbreviations (e.g., RR for relative risk, CI for confidence intervals
Provide information on nature of systematic review and standard steps*
Results, tables & figures
Define ‘no data available’ and reasons for empty cells
Define statistical terms*
Define relative risk and confidence interval in forest plots
Forest plots are difficult to understand
Provide interpretation of statistical results*
Define column labels
Avoid probabilities
Use similar table formats to aid readability with repeated exposure makes tables easier to read

## Slide 3
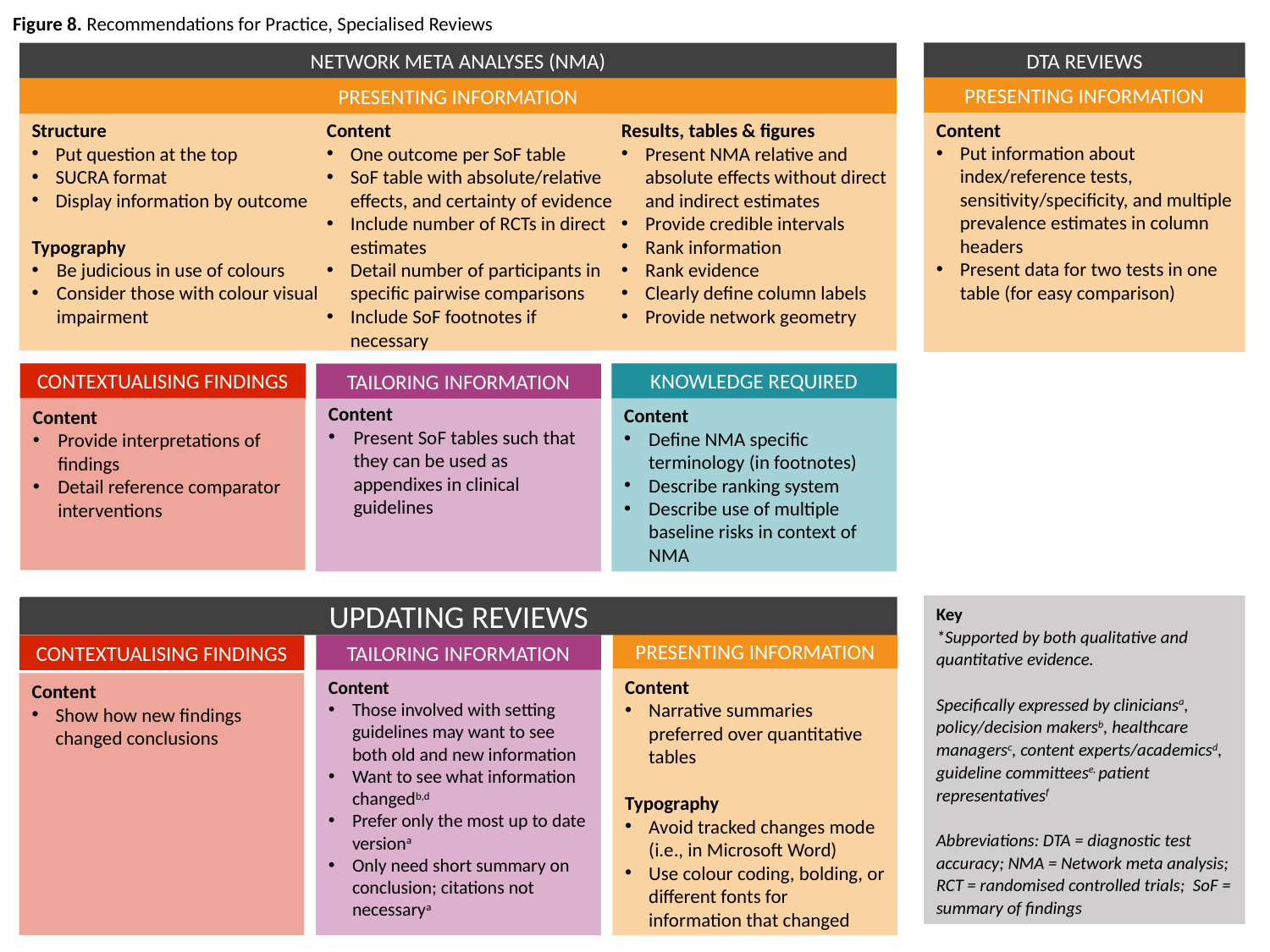

PRESENTING INFORMATION
PRESENTING INFORMATION
Figure 8. Recommendations for Practice, Specialised Reviews
DTA REVIEWS
NETWORK META ANALYSES (NMA)
Content
Put information about index/reference tests, sensitivity/specificity, and multiple prevalence estimates in column headers
Present data for two tests in one table (for easy comparison)
Structure
Put question at the top
SUCRA format
Display information by outcome
Typography
Be judicious in use of colours
Consider those with colour visual impairment
Content
One outcome per SoF table
SoF table with absolute/relative effects, and certainty of evidence
Include number of RCTs in direct estimates
Detail number of participants in specific pairwise comparisons
Include SoF footnotes if necessary
Results, tables & figures
Present NMA relative and absolute effects without direct and indirect estimates
Provide credible intervals
Rank information
Rank evidence
Clearly define column labels
Provide network geometry
CONTEXTUALISING FINDINGS
KNOWLEDGE REQUIRED
TAILORING INFORMATION
Content
Present SoF tables such that they can be used as appendixes in clinical guidelines
Content
Define NMA specific terminology (in footnotes)
Describe ranking system
Describe use of multiple baseline risks in context of NMA
Content
Provide interpretations of findings
Detail reference comparator interventions
PRESENTING INFORMATION
TAILORING INFORMATION
CONTEXTUALISING FINDINGS
Key
*Supported by both qualitative and quantitative evidence.
Specifically expressed by cliniciansa, policy/decision makersb, healthcare managersc, content experts/academicsd, guideline committeese, patient representativesf
Abbreviations: DTA = diagnostic test accuracy; NMA = Network meta analysis; RCT = randomised controlled trials; SoF = summary of findings
UPDATING REVIEWS
UPDATING REVIEWS
Content
Narrative summaries preferred over quantitative tables
Typography
Avoid tracked changes mode (i.e., in Microsoft Word)
Use colour coding, bolding, or different fonts for information that changed
Content
Those involved with setting guidelines may want to see both old and new information
Want to see what information changedb,d
Prefer only the most up to date versiona
Only need short summary on conclusion; citations not necessarya
Content
Show how new findings changed conclusions
