# Supplementary material for: The effectiveness and acceptability of evidence synthesis summary formats for clinical guideline development groups: a mixed-methods systematic review
Source: Implement Sci. 2022 Oct 27;17:74. doi: 10.1186/s13012-022-01243-2 (PMC9615384; doi:10.1186/s13012-022-01243-2)
Supplement: Supplementary file 7 — Additional file 7. Qualitative synthesis recommendations (with at least 3 supporting studies or mixed methods support). [file 13012_2022_1243_MOESM7_ESM.docx]

**Appendix 7.** Qualitative Synthesis Recommendations (with at least 3 supporting studies or mixed methods support)

| **Key:** * Supported by both qualitative and quantitative evidence. Specifically expressed by clinicians^a^, policy/decision makers^b^, healthcare managers^c^, content experts/academics^d^, guideline committees^e,^ patient representatives^f^  *For studies reporting on the same trial (i.e., Hartling 2016 and Hartling 2018* [1] *and Smith 2019 and Totten 2019* [2]*, only one paper is cited as to not visually misrepresent recommendations as being supported by more studies than it is)* |
| --- |

| **Presenting Information** |
| --- |
| **All reviews**  **Content**  First page   1. Give publication date [3–5] 2. Detail key messages [1,3,5–7]   General   1. Plain language [1,3,6,8–10] ~~and jargon-free~~ [4] 2. Avoid repetition [3,8,11] 3. Give PICOS information [8,10,12] ~~and characteristics of included studies [13]~~ 4. Rank evidence and recommendations [1,4,8] 5. Noticeable hyperlinks to supporting documents (full review, data, individual studies, etc.) [1–3,3,4,14]   **Structure**   1. Concise [3,4,8,9,11,15] 2. Brief narrative report [5,7]* 3. Structured [1,4,5,7,9]* 4. Use Summary of Findings (SoF) tables [3,6–8,11,13,16]*, ~~allowing for qualitative data [11] in prominent positions [12]~~   **Typography**   1. Use bullet points [3,8,17] 2. Avoid dense information. Promote white space [1,3,5]   **Results, tables & figures**   1. Decrease numeric/statistical data [3,9,12] 2. Present numbers in tables and/or visually [9]; use icons [3] or graphics [5]^b^[18] 3. Don’t break tables over multiple pages [3,6,10] |
| **Tailoring Information** |
| **All reviews**  **Content**   1. Choice and control over the amount of detail received [2–4] |
| **Contextualising Findings** |
| **All reviews**  **Content**   1. Framed within local [2,4,6], ~~national, [5,9] or broader~~ context ~~[6]~~^~~b~~^ 2. Implementation/application information [3,8,9] 3. Recommendations for practice/policies [2–4,6,12] ~~and future research needs [13]~~ 4. Effective intervention details [3,12] to help implementation, (e.g., dosages [3,8], trade names [8]^a^, treatment duration/frequency [3,8], costs [8]^a^, settings [3,14], evaluators of treatments [8]^a^, prevalence estimates [3,11], population characteristics [2,3,8,14] |
| **Quality of Evidence** |
| **Content**   1. Include quality assessment of evidence/study quality [1,2,9,10,13,19]* 2. Provide distinct explanations of rating scale (GRADE) [7–11,16,19,20]* 3. Detail how authors arrived at assessments of quality [5,7,19]* in footnotes [11] |
| **Trust in Producers and Summary** |
| **Content**   1. Include authors’ names [4,6,8,14] 2. Put logos on first page [1,4–6] |
| **Knowledge Required** |
| **Content**   1. Avoid field-specific or technical jargon (e.g., ‘scaling up’, ‘EBM’, ‘PICO’) [6,7,14] 2. Provide information on nature of systematic review and standard steps [6,13,14,18]*   **Results, tables & figures**   1. Define statistical terms [3,5,6,11,12,14,16]* 2. Provide interpretation of statistical results [7,16,18]* |

**References**

1. Hartling L, Gates A, Pillay J, Nuspl M, Newton AS. Development and Usability Testing of EPC Evidence Review Dissemination Summaries for Health Systems Decisionmakers. Agency for Healthcare Research and Quality (US); 2018; Available from: http://ovidsp.ovid.com/ovidweb.cgi?T=JS&PAGE=reference&D=medp&NEWS=N&AN=30507111

2. Totten AM, Smith C, Dunham K, Jungbauer RM, Graham E. Improving Access to and Usability of Systematic Review Data for Health Systems Guidelines Development. Agency for Healthcare Research and Quality (US); 2019; Available from: http://ovidsp.ovid.com/ovidweb.cgi?T=JS&PAGE=reference&D=medp&NEWS=N&AN=31013017

3. Marquez CJ Alekhya Mascarenhas; Jassemi, Sabrina; Park, Jamie; Moore, Julia E; Blaine, Caroline; Bourdon, Gertrude; Chignell, Mark; Ellen, Moriah E; Fortin, Jacques; Graham, Ian D; Hayes, Anne; Hamid, Jemila S; Hemmelgarn, Brenda R; Hillmer, Michael P; Holmes, Bev; Holroyd-Leduc, Jayna; Hubert, Linda; Hutton, Brian; Kastner, Monika; Lavis, John N; Michell, Karen; Moher, David; Ouimet, Mathieu; Perrier, Laure; Proctor, Andrea; Noseworthy, Tom; Schuckel, Victoria; Stayberg, Sharlene; Tonelli, Marcello; Tricco, Andrea C; Straus, Sharon E. Enhancing the uptake of systematic reviews of effects: what is the best format for health care managers and policy-makers? A mixed-methods study. 2018;13:84-NA.

4. Dobbins MT Helen; O’Brien, Mary Ann; Duggan, Melissa. Use of systematic reviews in the development of new provincial public health policies in Ontario. 2004;20:399–404.

5. Busert LK, Mütsch M, Kien C, Flatz A, Griebler U, Wildner M, et al. Facilitating evidence uptake: Development and user testing of a systematic review summary format to inform public health decision-making in German-speaking countries. Health Research Policy and Systems [Internet]. 2018;16. Available from: https://www.scopus.com/inward/record.uri?eid=2-s2.0-85049782278&doi=10.1186%2fs12961-018-0307-z&partnerID=40&md5=8a60b2081f09fd2655dac0ddecb23467

6. Rosenbaum SE, Glenton C, Wiysonge CS, Abalos E, Mignini L, Young T, et al. Evidence summaries tailored to health policy-makers in low- and middle-income countries. Bull World Health Organ. 2011;89:54–61.

7. Opiyo N, Shepperd S, Musila N, Allen E, Nyamai R, Fretheim A, et al. Comparison of Alternative Evidence Summary and Presentation Formats in Clinical Guideline Development: A Mixed-Method Study. PLoS ONE [Internet]. 2013;8. Available from: ://WOS:000315210400056

8. Perrier LK M Ryan; Straus, Sharon E. An iterative evaluation of two shortened systematic review formats for clinicians: a focus group study. 2014;21:e341-6.

9. Buljan I, Tokalić R, Roguljić M, Zakarija-Grković I, Vrdoljak D, Milić P, et al. Comparison of blogshots with plain language summaries of Cochrane systematic reviews: a qualitative study and randomized trial. Trials. 2020;21:426.

10. Yepes-Nunez JJ, Li SA, Guyatt G, Jack SM, Brozek JL, Beyene J, et al. Development of the summary of findings table for network meta-analysis. Journal of Clinical Epidemiology. 2019;115:1–13.

11. Mustafa R, Wiercioch W, Brozek J, Lelgemann M, Buehler D, Garg A, et al. Enhancing the acceptance and implementation of grade summary tables for evidence about diagnostic tests. BMJ Quality and Safety. 2013;22:A36.

12. Rosenbaum SG Claire; Nylund, Hilde Kari; Oxman, Andrew D. User testing and stakeholder feedback contributed to the development of understandable and useful Summary of Findings tables for Cochrane reviews. 2010;63:607–19.

13. Hartling L, Guise JM, Hempel S, Featherstone R, Mitchell MD, Motu’apuaka ML, et al. Fit for purpose: Perspectives on rapid reviews from end-user interviews. Systematic Reviews [Internet]. 2017;6. Available from: https://www.scopus.com/inward/record.uri?eid=2-s2.0-85013130994&doi=10.1186%2fs13643-017-0425-7&partnerID=40&md5=d0ea651ce13e9b75e702f2b5a9e822cc

14. Steele R. Mental health clinicians views of summary and systematic review utility in evidence-based practice. Health Information and Libraries Journal [Internet]. Available from: ://WOS:000627057300001

15. Babatunde OO, Tan V, Jordan JL, Dziedzic K, Chew-Graham CA, Jinks C, et al. Evidence flowers: An innovative, visual method of presenting “best evidence” summaries to health professional and lay audiences. Research Synthesis Methods. 2018;9:273–84.

16. Rosenbaum SG Claire; Oxman, Andrew D. Summary-of-findings tables in Cochrane reviews improved understanding and rapid retrieval of key information. 2010;63:620–6.

17. Newberry SJ, Shekelle PG, Vaiana M, Motala A. Reporting the Findings of Updated Systematic Reviews of Comparative Effectiveness: How Do Users Want To View New Information? Agency for Healthcare Research and Quality (US); 2013; Available from: http://ovidsp.ovid.com/ovidweb.cgi?T=JS&PAGE=reference&D=medp&NEWS=N&AN=23785728

18. Buljan I, Malički M, Wager E, Puljak L, Hren D, Kellie F, et al. No difference in knowledge obtained from infographic or plain language summary of a Cochrane systematic review: three randomized controlled trials. J Clin Epidemiol. 2018;97:86–94.

19. Carrasco-Labra A, Brignardello-Petersen R, Santesso N, Neumann I, Mustafa RA, Mbuagbaw L, et al. Improving GRADE evidence tables part 1: a randomized trial shows improved understanding of content in summary of findings tables with a new format. Journal of Clinical Epidemiology. Elsevier; 2016;74:7–18.

20. Perrier LK M Ryan; Straus, Sharon E. A usability study of two formats of a shortened systematic review for clinicians. 2014;4:e005919-NA.
